# Nanotechnology for Targeted Detection and Removal of Bacteria: Opportunities and Challenges

**DOI:** 10.1002/advs.202100556

**Published:** 2021-09-23

**Authors:** Mohammad J. Hajipour, Amir Ata Saei, Edward D. Walker, Brian Conley, Yadollah Omidi, Ki‐Bum Lee, Morteza Mahmoudi

**Affiliations:** ^1^ Department of Radiology and Precision Health Program Michigan State University East Lansing MI 48824 USA; ^2^ Division of Physiological Chemistry I Department of Medical Biochemistry and Biophysics Karolinska Institutet Stockholm 171 65 Sweden; ^3^ Department of Entomology Michigan State University East Lansing MI 48824 USA; ^4^ Department of Microbiology and Molecular Genetics Michigan State University East Lansing MI 48824 USA; ^5^ Department of Chemistry and Chemical Biology Rutgers The State University of New Jersey Piscataway NJ 08854 USA; ^6^ Department of Pharmaceutical Sciences College of Pharmacy Nova Southeastern University Fort Lauderdale FL 33328 USA

**Keywords:** bacteria, biofilms, nanotechnology, resistance

## Abstract

The emergence of nanotechnology has created unprecedented hopes for addressing several unmet industrial and clinical issues, including the growing threat so‐termed “antibiotic resistance” in medicine. Over the last decade, nanotechnologies have demonstrated promising applications in the identification, discrimination, and removal of a wide range of pathogens. Here, recent insights into the field of bacterial nanotechnology are examined that can substantially improve the fundamental understanding of nanoparticle and bacteria interactions. A wide range of developed nanotechnology‐based approaches for bacterial detection and removal together with biofilm eradication are summarized. The challenging effects of nanotechnologies on beneficial bacteria in the human body and environment and the mechanisms of bacterial resistance to nanotherapeutics are also reviewed.

## Graphical abstract

Nanotechnologies are being used in the identification, discrimination, and removal of a wide range of pathogens. Pathogens, on the other hand, evolve over time and use different lines of defense and strategies to protect themselves against new treatments including nanotechnologies.

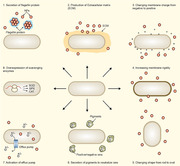

## 1 Introduction

**Figure 1:**
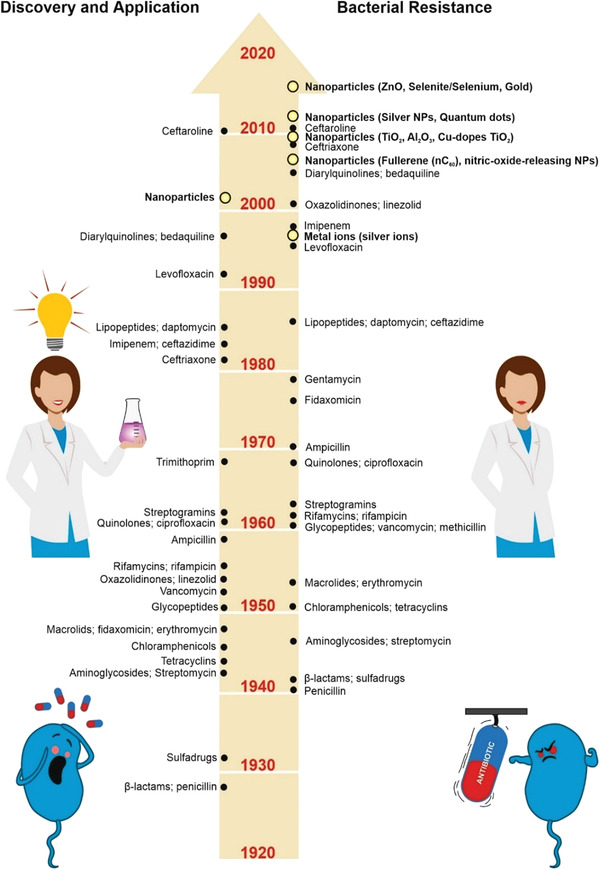
Descriptive timeline of bacterial resistance to antibiotics and NPs.^[^ ^6^, ^10^ ^]^

Bacterialinfection is a growing healthcare concern mainly due to the current misbalancing between the discovery of new drugs and bacterial resistance process rates (**Figure** **1** summarizes the timeline of bacterial resistance to antibiotics).^[^ ^1^ ^]^ Therefore, there is an urgent need to develop new antibacterial therapeutics to address these growing concerns. In the past few decades, nanotechnologies have been increasingly developed and used to address the bacterial resistance issue with promising outcomes.^[^ ^2^ ^]^ Nanoparticles (NPs) have a capacity to interact with bacterial membranes and cause disruption of efflux pumps and membrane integrity together with induction of oxidative stress.^[^ ^3^ ^]^ Unlike conventional antibiotics, NPs can pass biological and biofilm barriers (e.g., by using an external magnetic field on magnetic NPs).^[^ ^4^ ^]^ NPs are also able to effectively kill bacteria, before growing and infection development, by inhibiting the density‐dependent cell–cell signaling triggering bacterial growth, virulence, and resistant.^[^ ^5^ ^]^ However, some bacteria demonstrated inherent resistance to NPs or became resistant to even a high concentration of NPs after repeated long‐time exposure to NPs (see Figure 1 for details).^[^ ^6^ ^]^ Therefore, the development of precision antibacterial therapeutics is a rational strategy to use the lower NP concentration with the highest therapeutic efficacy to kill bacteria in the shortest possible period.

The development of sensory NPs for sensitive, robust, and early detection of pathogens is another extensive contribution of nanotechnologies to the bacterial infection issue.^[^ ^7^ ^]^ Identification and discrimination of pathogens at low concentrations are of great clinical importance as such knowledge enables healthcare providers in determining the right antibiotic medications to prevent consequences of bacterial infection (e.g., in infectious chronic wounds^[^ ^8^ ^]^).

This review will cover the application, effect, and mechanisms of nanotechnology for antibacterial applications, focusing primarily on the use of NPs with varying compositions and physicochemical properties. Furthermore, this review will describe how bacterial communities’ bacteria respond to nanotechnology‐based approaches at a genetic and population‐based level. We also summarize the adverse effects of NPs on beneficial bacteria in our body and environment, which may cause serious human health and eco‐environmental problems.^[^ ^9^ ^]^

## 2 Nanotechnology‐Based Bacteria Detection

The sensitive, selective, and rapid detection of food/water/air‐borne infections and clinical pathogens is a critical step in the prevention/control of pathogenic outbreaks, treatment of bacterial infections, and environmental safety/monitoring. Conventional bacterial diagnostic approaches such as bacterial culture, morphologic analysis, biochemical staining, enzyme‐linked immunosorbent assay, and polymerase chain reaction are time consuming and entail complex pretreatment procedures, preparation/enrichment of samples, advanced analytical equipment, and skillful technicians.^[^ ^11^ ^]^ There is, therefore, an urgent need to develop new sensors that address the current challenges in detection and discrimination of even low concentrations of bacteria, with high specificity and sensitivity, in a short period of time (e.g., a few minutes to a few hours). Several biosensors and probes with a wide range of recognition elements (e.g., aptamers, antibodies, enzymes, DNA, and bacteriophage) possessing such properties have been developed.^[^ ^12^ ^]^

**Figure 2:**
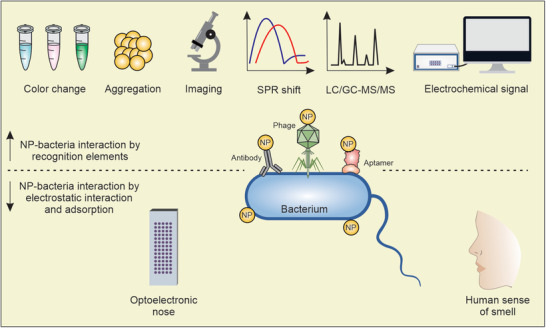
Schematic representation of nanotechnology‐based diagnostic approaches developed for bacterial detection and discrimination. a) The specific binding of NPs to bacteria is mediated by recognition elements (e.g., antibody, phage, or aptamer), and then the NP–bacteria complex is monitored based on the changes in the color of the solution, NP aggregation, surface plasmon resonance shift, mass spectrometry peaks, and electrochemical signals. b) The nonspecific binding of NPs to bacteria was also subjected to detect and discriminate different bacterial species. The array‐based sensors mimic the human olfactory system, generate specific array responses that serve as fingerprints for bacterial species. Optomechanical resonators detect and discriminate even a single bacterium based on bacterial distinct vibration mode.

The application of NPs to the development of biosensors has produced diagnostic tools for bacterial detection (**Figure** **2**a,b and **Table** **1**).

**Table 1:** Nanotechnology‐based approaches for detection and discrimination of bacteria

NP composition	NP type	Identified bacteria	Mechanisms and/or methods of detection	Recognition element	Advantages versus shortcomings	Size of NP [nm]	Remark	Ref.
Gold magnetite nanocomposites	Metal	*S. aureus*	Plasmon absorbance, fluorescence, and optical and confocal images	Anti‐*α*‐toxin antibody	Concurrent detection of a *S. aureus* based on different approaches (plasmon absorbance, fluorescence/confocal and optical microscopy).	85–100	NPs targeted *α*‐toxin specifically expressed on *S. aureus*. Detection sensitivity: 102 CFU mL^−1^ Detection time: 30 min	^[^ ^52^ ^]^
Protein‐A‐modified silver NPs	Metal	*E. coli*	Surface‐enhanced Raman scattering	Polyclonal antibodies	This diagnostic approach can be used for a few number of bacteria. The intensity was considerably enhanced (20‐fold) compared to conventional Raman spectrum.	70–100	*E. coli* was specifically detected even in the presence of *Rhodococcus rhodochrous*.	^[^ ^53^ ^]^
Zeolitic imidazolate framework/gold NP composite	Metal	*A. hydrophila* and *P. aeruginosa*	Sandwich immunoassay	Thionine‐labeled anti‐*A. hydrophila* and ferrocene‐labeled anti‐ *P. aeruginosa*	This diagnostic approach has low detection limit and can simultaneously detect *P*. *aeruginosa* and *A. hydrophila* in various food samples.	200	Detection sensitivity: *P. aeruginosa* 8.09 CFU mL^−1^ *A. hydrophila* 3.60 CFU mL^−1^	^[^ ^20^ ^]^
Streptavidin‐coated Au NPs	Metal	*E. coli* and *Legionella pneumophila*	LSPR signal enhancement	Biotinylated antibacteria antibody	‐ Enhancement of LSPR signal ‐ Maximized limit of detection ‐ Maximized bacterial detection sensitivity and rapidity	5–50	Detection sensitivity: *E. coli* and *L. pneumophila* 10^2^ and 10^1^ CFU mL^−1^	^[^ ^17^ ^]^
Polyalanine‐coated magnetic NPs and gold NPs	Metal	*E. coli O157:H7*	Electrochemical sensing of gold NP as probe	Monoclonal and polyclonal antibodies	‐ Rapid detection of pathogen ‐ Maximized detection limit ‐ Sample preparation needs only an external magnetic field	50–100	Detection sensitivity: 10^1^–10^6^ CFU mL^−1^	^[^ ^21^ ^]^
Gold NPs	Metal	T7 bacteriophage	NP aggregation and color change from red to purple	Anti‐T7 antibodies	NP aggregation and consequent color change is detected by naked eye. This approach is flexible and can be easily fitted for detection of other types of viruses and bacteriophages.	30	Detection sensitivity: 1.08 × 10^10^ PFU mL^−1^	^[^ ^19^ ^]^
Magnetic NP	Metal	*Cryptosporidium parvum*	Dark‐field microscopy imaging	Anti‐*C. parvum* polyclonal IgG antibody	‐ This approach is fast, user friendly, and cheap ‐ Maximized limit of detection	≈800	Detection sensitivity: 8.7 oocysts	^[^ ^54^ ^]^
Mesoporous‐TiO_2_‐coated magnetic NP	Metal	*E. coli* and *S. aureus*	Fluorescent imaging	Aptamer	Fast and sensitive detection of pathogenic bacteria in bloodstream. This approach is flexible and can be easily fitted for detection of other types of pathogens.	200	Detection sensitivity: 10–2000 CFU mL^−1^	^[^ ^26^ ^]^
Silver NPs And streptavidin‐coated magnetic beads	Metal	*S. aureus*	Sandwich immunoassay (electrochemical immunosensor)	Biotinylated primary anti‐*S. aureus* aptamer Secondary anti‐*S. aureus* aptamer	Sandwich assay advantages are simplicity, rapidity, and high specificity.	20	Detection sensitivity: 10–10^6^ CFU mL^−1^	^[^ ^55^ ^]^
Gold NP	Metal	*S. typhimurium*	LSPR signal sensing	Aptamer	Higher sensitivity and specificity for detection of pathogen in pork meat without pre‐enrichment. Food matrix or other contaminant flora did not affect the accuracy and performance of this nanosensor.	20	Detection sensitivity: 10^4^ CFU mL^−1^	^[^ ^56^ ^]^
Gold NP	Metal	*S. aureus*	Resonance light‐scattering signal	SA17 and SA61 aptamers	This nanosensor is ultrasensitive and detects single *S. aureus* cell.	60	Single *S. aureus* cell	^[^ ^27^ ^]^
Gold nanorod magnetic NPs	Metal	*E. coli O157:H7*	Surface‐enhanced Raman scattering	Primary aptamer (Apt‐1) Secondary aptamer (Apt‐2)	‐ High sensitivity This nanosensor can be used for detection of food pathogens.	The GNRs: (52 × 22) Magnetic NP: 150	Detection sensitivity: 3 CFU mL^−1^	^[^ ^24^ ^]^
Thiolated gold NPs	Metal	*E. coli*, *P. aeruginosa*, *V. cholerae*, *X. campestris*	NP aggregation and color change Shift in SPR peak	The RBPs of genus *Inovirus* were expressed on M13 phage and used as recognition element.	This nanosensor can detect bacteria in sea water and serum and has potential clinical application.	4	Detection sensitivity: 10^2^ CFU mL^−1^	^[^ ^31^ ^]^
Alumina‐coated magnetic NPs	Metal	*A. baumannii* M3237 and 54149	Mass spectrometry	Tail fibers 2 and 6 displayed in ɸAB2 and ɸAB6 phages, respectively	This nanosensor is ultrasensitive and distinguishes *A. baumannii* M3237 from *A. baumannii* 54149		Detection sensitivity: *A. baumannii* M3237 10^5^ cells mL^−1^ *A. baumannii* 54149 10^4^ cells mL^−1^	^[^ ^34^ ^]^
SiO_2_@Au core–shell NPs	Metal	*S. aureus*	Dark‐field microscopy imaging	Phage S13′	‐This nanosensor specifically detected *S. aureus* in the presence of other bacteria such as *E. coli*. ‐This approach is flexible and can be easily fitted for detection of any bacterium by changing the phages employed.	557	Detection sensitivity: 8 × 10^4^ CFU mL^−1^	^[^ ^35^ ^]^
Albumin‐templated Co_3_O_4_ magnetic nanozymes	Metal	*S. aureus*	Magnetophoretic chromatography and color change using the catalytic oxidation of 2,2′‐azino‐bis(3‐ethylbenzo‐thiazoline‐6‐sulfonic acid).	Phage fusion pVIII protein	Detection of *S. aureus* in complex media such as milk.	210	Detection sensitivity: 8 CFU mL^−1^	^[^ ^57^ ^]^
Gold–silver alloy nanocluster	Metal	13 types of sulfur‐oxidizing bacteria; sulfur‐containing bacteria	Light‐scattering signal, fluorescence, UV–vis absorbance,	–	Identification and discrimination of sulfur‐oxidizing bacteria and nonsulfur bacteria.	33–130	Sulfur containing/oxidizing species show different affinities to gold–silver alloy nanocluster. Detection time: 30 min	^[^ ^7d^ ^]^
Cationic gold NPs (modified amine headgroup)	Metal	*S. aureus* *E. coli*	Electrochemical sensing of *β*‐galactosidase enzyme activity.	–	‐ Sensitive, fast, and simple This nanosensor is not able to detect bacteria in multiplex systems with strong background.	≈2	Detection sensitivity: 10^2^ CFU mL^−1^ Detection time: <60 Min	^[^ ^49b^ ^]^
Cationic gold NPs	Metal	*E. coli*, *B. subtilis*, *Micrococcus luteus*, *P. aeruginosa*	Human sense of smell of generated scent	–	‐ Bacterial detection without any instrument ‐ Sensitive and fast	≈2	Detection sensitivity: 10^2^ CFU mL^−1^ Detection time: 15 min	^[^ ^47^ ^]^
Different gold NPs coated with mercaptopropionic acid, mercaptosuccinic acid, cysteamine, and cetyltrimethylammonium bromide	Metal	*S. aureus*, *S. epidermidis*, *L. monocytogenes*, *B. aceticus*, *P. aeruginosa*, *E. coli*, *B. subtilis*, *S. paratyphi*, *Vibrio parahemolyticus*, *Clostridium putrefaciens*	NP aggregation and color change	–	Color shift could be detected by naked eye. This approach is ultrafast and discriminates distinct types of bacteria in microorganism mixtures.	15–60	Detection time: 5 s	^[^ ^36b^ ^]^
Cationic gold NPs (modified amine headgroup)	Metal	*E. coli*	Color change and colorimetric readout	–	‐ Rapid and sensitive ‐ This nanosensor showed different sensitivities to detect different types of bacteria	≈2	Detection sensitivity: 10^2^ bacteria mL^−1^ in solution	^[^ ^49a^ ^]^
SWCNT–gold NP nanohybrids	Semiconductor (carbon‐based‐) metal	*S. typhimurium DT104*	Surface‐enhanced Raman scattering	Rhodamine 6G (Rh6G)‐modified monoclonal AC04 antibody	Enhancement of Raman signal intensity. This nanosensor specifically detects *Salmonella DT104* and distinguishes between different types of bacteria.	20	Detection sensitivity: 105 CFU mL^−1^ This nanohybrid selectively detected and killed *S. typhimurium DT104* over 15 min.	^[^ ^58^ ^]^
Trimethyl chitosan NPs	Polymer	*E. coli (DH5a)*.	Fluorescence intensity	Single‐strand DNA aptamer	This nanosensor only detects high concentration of bacteria.	323 ± 10	Detection sensitivity: 10^5^ CFU mL^−1^	^[^ ^25^ ^]^
Magnetic bead coated by positively charged polymer	Metal–polymer	*P. mirabilis* and *P. aeruginosa*	NP aggregation and color change (redshift LSPR)	–	‐ Ultrafast ‐ Ultrasensitive ‐ Identification of urease‐positive bacteria in urine with minimal instrument ‐ Detection of color changes by naked eye	38	Detection sensitivity: 10^1^ cells mL^−1^ Detection time: 40 min	^[^ ^39^ ^]^
Hyaluronic acid NPs loaded with four derivatives of 3‐hydroxyflavone	Biological NPs	*S. aureus*, *S. epidermidis*, *Bacillus subtilis*, *Enterococcus faecalis*, *E. coli*, *Acinetobacter baumannii*, *Klebsiella pneumoniae*, *Citrobacter freundii*	Ratiometric fluorescent analysis	–	This diagnostic approach is flexible, and expandable to detect other types of bacteria. This diagnostic approach is not able to recognize components in mixed systems.	–	Ratiometric fluorescent sensor array discriminated 8 bacterial species	^[^ ^59^ ^]^

PFU, plaque‐forming units; IgG, Immunoglobulin G; CFU, colony forming unit.

For example, plasmonic nanomaterials such as gold and silver NPs, which have surface plasmon resonance (SPR) properties, are widely used for bacterial detection.^[^ ^13^ ^]^ Once modified with recognition elements (e.g., antibody, phage, or aptamer), they are used for detecting particular bacteria. After specific binding to bacteria (with a sensitivity/detection range for a visible change: 10^3^–10^6^ bacteria), mediated by targeting ligands/recognition elements, NPs show plasmon peak shift or aggregation, leading to color changes visible to the naked eye. The specific binding of NPs to bacteria is also monitored using optical imaging, electrochemical sensing, and spectrometry.^[^ ^13^, ^14^ ^]^ In addition, label‐free array‐based sensors have also been developed to detect and discriminate bacteria and/or analytes. They consist of cross‐reactive indicators that preferentially interact with bacteria/analytes. The array‐based sensors mimic the human olfactory system, producing distinctive patterns of array response that serve as unique optical/electrical fingerprints for bacterial species. Ultrahigh frequency optomechanical resonators are a new generation of sensors developed to detect a single bacterium based on bacterial vibration mode.^[^ ^15^ ^]^ Here, we discuss recent advances in nanotechnology‐based approaches for bacterial detection.

### 2.1 Immune‐Based Sensors

NPs are mainly modified with targeting species (e.g., antibodies) as their recognition element, to allow them to target and attach to receptors/proteins/epitopes expressed on the surface of the target bacteria. This ligand–receptor interaction induces NP aggregation of the nearby targeted pathogen and changes the SPR/colorimetric response.^[^ ^16^ ^]^ SPR is a unique property of metallic NPs. The oscillation of electron clouds in metallic atoms within NPs is sensitive to surface binding events and allows researchers to carefully monitor NP–analyte interactions. As such, detection based on color change is the most popular approach for the immune sensing of bacteria. For example, the aggregation of antibody‐conjugated gold NPs after specific interaction with targeted bacteria is widely used to detect a broad range of bacteria. Gold NPs functionalized with antibodies specific to *Escherichia coli* and *Legionella pneumophila* aggregated to produce a localized SPR (LSPR) color change from red to purple when attached to these bacteria.^[^ ^17^ ^]^

Recognition elements such as antibodies have high affinity and avidity to their corresponding antigens expressed on bacteria; therefore, the best way to improve the sensitivity of immunosensors is to enhance the LSPR signal or promote the detection of SPR signals that are representative of binding target bacteria. Depending on the size, shape, particle and binding site number, and aggregation state of gold NPs, NP‐based colorimetric sensors show different sensitivities and performances. For example, in a study utilizing streptavidin‐coated Au NPs from sizes ranging from 5 to 50 nm, Au NPs with fewer and larger particles showed higher sensitivity and selectivity.^[^ ^17^ ^]^ As many types of antibodies targeting a large number of pathogens are available, and the surface functionalization of gold NPs is feasible, the design of colorimetric immune sensors is adaptable to isolate many types of pathogens. Biosensing based on the LSPR color change approach is also used to detect *Salmonella typhimurium DT104*.^[^ ^18^ ^]^ Gold NPs functionalized with a monoclonal antibody sensitively and selectively to detect and discriminate *S. typhimurium DT104* (10^3^ CFU g^−1^ or above) from other genus and species on a romaine lettuce that were infected by mixture of bacteria such as *Salmonella* ser. *Agona*, *E. coli*, and *S. typhimurium DT104* changed the color of the NP solution from purple to gray.

In addition to bacterial pathogens, colorimetric immunosensors were also developed to detect bacteriophages. A gold NP covalently modified with an anti‐T7 antibody specifically captured T7 phage, as a model of adenovirus, and rapidly aggregated, leading to a red‐to‐purple change. No aggregation or color change was seen when NPs were exposed to M13 phage and phosphate‐buffered saline solutions. Therefore, the colorimetric immunosensor can be used for sensitive and selective detection of different genera and/or species of pathogens.^[^ ^19^ ^]^

Different types of bacteria can be detected depending on the type of antibodies immobilized on the NP or platform–biosensor surface. A sandwich immune assay was designed for the identification of both *Aeromonas hydrophila* and *Pseudomonas aeruginosa*. Antibodies specific to each were immobilized on a substrate consisting of a zeolitic imidazolate framework (ZIF)‐8 and gold NP and used for real‐time detection of the two bacteria. Due to the increased surface area with Au NPs, a high concentration of antibodies was conjugated to the surface and resulted in a lower detection limit of the ZIF‐8/Au platform.^[^ ^20^ ^]^ Using a novel strategy, an immune‐based electrochemical biosensor was developed to purify *E. coli O157:H7* using magnetic and gold NPs. First, the magnetic NP functionalized with monoclonal antibodies selectively captured *E. coli O157:H7*. Then, the gold NPs modified with polyclonal antibodies were added to the solution to identify the bacteria.^[^ ^21^ ^]^ After magnetic separation of the complex, the identity and quantity of captured bacteria were assessed by monitoring the electrochemical signals of gold NPs.

### 2.2 Aptasensors

Although monoclonal antibodies have sufficient sensitivity and selectivity for bacterial recognition, their purification and production are expensive and time consuming. In addition, issues with solubility and stability remain to be addressed. Therefore, alternative biorecognition elements have been used for the development of nanosensors.

Aptamers are short sequences of peptide or single‐strand DNA or RNA (20 to 60 nucleotides) that can fold into different structures and target bacteria, proteins, and enzymes with high affinity and specificity.^[^ ^22^ ^]^ Nucleotide alternatives to antibodies are inexpensive, readily modifiable with reporters, and show neither immunogenicity nor toxicity even at high doses. Aptamers can be designed straightforwardly via systematic evolution of ligands by exponential enrichment, enabling the identification of a matching sequence from a vast database of random sequences to target many different types of receptors/epitopes expressed explicitly on the bacterial species.^[^ ^23^ ^]^ Aptamers are widely used as recognition elements in surface‐enhanced‐Raman‐scattering (SERS)‐ and fluorescent‐based sensors developed for bacterial detection.^[^ ^24^ ^]^ For example, fluorescent trimethyl chitosan NPs functionalized with an aptamer of a high affinity to *E. coli* (DH5a), selectively bound to the bacteria. The concentration and identity of NP‐bound bacteria were then determined based on fluorescence intensity.^[^ ^25^ ^]^

An aptamer‐based approach utilizing TiO_2_‐coated magnetic NP conjugated to aptamers targeting bacterial bloodstream infections (BSI) successfully isolated, enriched, and identified BSI such as *E. coli* and *Staphylococcus aureus*.^[^ ^26^ ^]^ Bacteria were captured through the aptamer interacting with the corresponding epitope on the bacteria. The aptamer could specifically bind to the chosen target molecule through folding into a sequence‐defined unique structure. The captured bacteria were then enriched and isolated using an external magnetic field. *E. coli* and *S. aureus* were thus captured, separated, and concentrated in only 2 h; significantly faster than traditional assays. This versatile platform can be used to isolate and detect many types of bacteria by selecting an aptamer for the epitope of interest.

A dual‐aptamer‐based sandwich immunosensor was developed for selective detection of *S. aureus*.^[^ ^27^ ^]^ Primary aptamer conjugated to a magnetic bead recognized and captured the *S. aureus*. Then, the secondary aptamer conjugated to a silver NP (as a probe) was added to the solution, and the resulting complex was isolated by an external magnetic field. The identity and concentration of bacteria were assessed by analysis of electrochemical signals generated by silver NPs. The high sensitivity and selectivity of this electrochemical aptasensor were derived from the specificity of the two aptamers used for “sandwiching” *S. aureus*.

In a similar strategy, a SERS aptasensor was developed to separate and detect *E. coli O157:H7*.^[^ ^24^ ^]^ Combining aptamer‐conjugated NPs with SERS is a promising strategy to detect pathogens. Gold NPs functionalized with an aptamer can specifically bind to and enhance the Raman signal of bacteria, due to localized SPR at the interaction site. Specific Raman spectrum changes/enhancement of bacteria after interaction with NPs are monitored to detect and discriminate different bacterial species. To separate and detect *E. coli O157:H7*, the magnetic NPs modified with secondary aptamer (Apt‐2), as a capturing agent, were incubated with *E. coli O157:H7*. Then, gold nanorods that served as an optimal, active SERS substrate, were modified with rhodamine and aptamer (Apt‐1) and added to the solution. Apt‐1 and Apt‐2 mediated the specific interaction of magnetic NPs and gold nanorods (respectively) with bacteria. The resulting complex containing bacteria, magnetic NPs, and gold nanorods was separated using a magnetic field and detected via signal probe analysis with a limit of detection of 3 CFU mL^−1^.

### 2.3 Bacteriophage‐Based Sensors

Although many researchers use aptamers as recognition elements, aptasensors developed for diagnostic purposes were less effective than had been hoped due to limitations such as poor stability/half‐life in biological mediums, possible secondary structure formation in larger nucleic‐acid‐based aptamers rendering issues with analyte specificity, and possible cross‐reactivity between analytes.^[^ ^28^ ^]^

Bacteriophages are capable not only of expressing proteins bound to bacterial receptors, but can also recognize live and dead cells and proliferate in live cells.^[^ ^29^ ^]^ They are cheap, easily produced, and infect only bacteria. Moreover, bacteriophages demonstrate enhanced stability compared to their antibody counterparts, which are more sensitive to environmental stress (i.e., pH, temperature, etc.).^[^ ^30^ ^]^ Genetic engineering can also produce distinct types of bacteriophages or receptor binding proteins (RBPs) that specifically target the desired bacterial species. Through genetic engineering, RBPs for the genus *Inovirus* were expressed on M13, as a phage scaffold, and the resulted chimeric phage was used to detect the desired bacterial species.^[^ ^31^ ^]^ Compared to the native phage (i.e., bacteriophage with no engineered RBP), the M13 scaffold has fewer adverse consequences and greater proliferation in bacteria. The *Inovirus* RBPs can target a wide range of gram‐negative bacteria. The N‐terminus of this protein binds to bacterial pili while its C‐terminus attaches to the bacterial receptor and facilitates phage entrance.^[^ ^32^ ^]^ The M13 phage scaffold expressing RBPs was thiolated to be conjugated onto gold NPs, incubated with bacteria, and then gold NPs were added to the solution. The M13 phage acted as a bridge specifically mediating the interaction of bacteria and gold NPs, simultaneously attaching to them through RBPs and a thiol group, respectively. As a result of the gold NPs’ aggregation stemming from interaction with thiolated M13 phage, the solution color changed from red to blue. This colorimetric sensor array detected *E. coli*, *P. aeruginosa*, *Vibrio cholerae*, and *Xanthomonas campestris* with high sensitivity (100 cells) and specificity.^[^ ^31^ ^]^ In a similar study, gold NPs coated with T4 and BP14 bacteriophages specifically detected *E. coli* and methicillin‐Resistant *S. aureus* (MRSA) (respectively) based on SPR changes.^[^ ^33^ ^]^

Tail fibers (TF) of bacteriophages can also be genetically engineered and used for targeting bacterial species.^[^ ^34^ ^]^ The recombinant TF2 and TF6 displayed in ɸAB2 and ɸAB6 can specifically attach to the bacteria walls of *Acinetobacter baumannii* M3237 and 54149, respectively. *A. baumannii* is an opportunistic bacterium that is a frequent culprit of nosocomial infection in hospital settings. Both TFs were tagged with hexahistidine and separately linked to alumina‐coated magnetic NPs. The magnetic core was used to separate the bacteria by an external magnetic field. The synthesized TF6–Fe_3_O_4_@Al_2_O_3_ and TF2–Fe_3_O_4_@Al_2_O_3_, which were specifically attached to their corresponding receptors on the desired bacteria walls in a mixed bacterial solution, were separated using a magnetic field and subjected to mass spectrometry. TF‐immobilized magnetic NPs are promising candidates for the separation and identification of the different bacterial strains.

Phage‐immobilized NPs enhanced bacterial detection and discrimination in dark‐field microscopy, the most sensitive and effective light‐scattering imaging tool for bacterial detection. A plasmon‐scattering nanoprobe was developed through immobilization of phage S13ʺ on the surface of SiO_2_@Au core–shell NPs. Phage S13ʺ mediated the selective binding of NP to *S. aureus* in the bacterial mixture. Under the dark‐field microscope, the plasmon scattering nanoprobes, specifically attached to *S. aureus* and consequently aggregated, showed higher scattering intensity than the few monodispersed probes randomly attached to *E. coli*.^[^ ^35^ ^]^

### 2.4 Array‐Based Sensors

Although biorecognition elements increase the sensitivity and selectivity of sensors, they regularly require complex design work, production, and functionalization procedures to guarantee reproducible and reliable operation; and they suffer from both instability and toxicity. Therefore, array‐based sensing, without the need for any recognition element or molecular labels, was developed to detect and discriminate bacteria and analytes.^[^ ^36^ ^]^ As bacterial species or analytes have different affinities to varying plasmonic NPs with different sizes, compositions, and surface chemistry, they show different levels of light scattering, fluorescent emission, or UV absorbance signal after exposure to plasmonic NPs.^[^ ^7d^ ^]^ Based on those characteristics, gold–silver alloy nanoclusters were developed for the detection and discrimination of sulfur‐producing bacteria. Due to their distinct affinities for gold–silver alloy nanoclusters, bacteria producing different types of sulfur created different optical patterns (3D signal readouts) at a concentration lower than 0.5 × 10^−6^ m. This 3D sensor array also effectively discriminated sulfur‐oxidation bacteria from other bacteria.^[^ ^7d^ ^]^

Sensing the activity of enzymes produced by bacteria is another interesting approach to detect bacterial species.^[^ ^37^ ^]^ Urease is an ideal candidate since it is not expressed in human cells. After activation, urease produces NH_3_, which alkalizes the local microenvironment and can affect the aggregation of nano‐/microparticles that are sensitive to local pH changes based upon surface chemistry.^[^ ^38^ ^]^ A plasmonic sensor array was developed to detect urease‐positive and ‐negative bacteria such as *Proteus mirabilis* and *P. aeruginosa*, respectively.^[^ ^39^ ^]^ This sensor array consisted of magnetic beads coated with a positively charged polymer, mediating the electrostatic interaction with negatively charged bacteria. The magnetic bead–bacteria complex rapidly aggregated in the presence of bovine serum albumin at acidic pH. Because the urease‐negative bacteria bound to magnetic beads cannot produce urease or alkalize the solution, magnetic beads aggregate, and the solution color changes from red to blue. This plasmonic nanosensor detects a low concentration (limit of detection of 10 cells mL^−1^) of urease‐positive/‐negative bacteria in a short period of time (40 min).

### 2.5 Optoelectronic Nose

The optoelectronic nose is an array‐based bacterial‐detecting method consisting of cross‐reactive sensors that interact with bacteria or their analytes and generate a unique pattern response, in essence, a distinct optical “fingerprint” for different bacteria.^[^ ^36^, ^40^ ^]^ It can act as an olfactory system by enabling bacterial detection based on a generated array response and by collating to a predefined library of responses.^[^ ^40a^ ^]^ A colorimetric sensor array consisting of 73 indicators was used to detect *Mycobacterium tuberculosis* in urine samples.^[^ ^41^ ^]^ Each indicator interacted specifically with volatile organic compounds (VOCs) emitted by bacteria and caused a distinct combination of color changes that can be used as a fingerprint to diagnose analytes. Using this approach, infection‐specific VOCs emitted by the urine of patients with tuberculosis were sensed. The accuracy and efficacy of this sensor were improved by chemical pretreatment of urine samples to increase the generation of VOCs. In a similar study, authors detected and discriminated 15 bacterial species cultured in solid agar media.^[^ ^42^ ^]^ A cross‐responsive colorimetric sensor array consisting of 36 indicators was developed to sense the VOCs generated by a mixture of bacteria grown in Petri dishes.^[^ ^43^ ^]^ The chemically responsive dyes used were highly sensitive to a wide range of VOCs and generated distinct fingerprint arrays for bacterial detection. Another colorimetric sensor array with 80 indicators specifically detected dangerous bacterial species even at extraordinarily low density (even at 8 or 9 CFU);^[^ ^44^ ^]^ it identified and differentiated *Bacillus anthracis* (8–11 CFU) and *Yersinia pestis* (9–50 CFU) with excellent accuracy. The optoelectronic nose is a disposable sensor that offers all the features required for sensitive and specific identification of bacteria. However, it does have disadvantages such as color shifting/fading over time and the necessity of collecting vast amounts of detection data to generate VOC patterns for collating bacterial recognition. In addition, postdetection analysis of VOC patterns may require extensive analytical procedures that may not be suitable for rapid detection. Therefore, semiquantitative and specific sensing of VOCs generated by bacteria are the main issues that should be addressed in developing the new generation of colorimetric VOC sensors.^[^ ^45^ ^]^

The human olfactory system can differentiate many odors with high sensitivity and specificity.^[^ ^46^ ^]^ A new generation of smell‐based sensor platforms has been developed to detect bacteria by a method called headspace analysis, which analyzes volatile compounds without directly contacting the sample.^[^ ^47^ ^]^ In one report, cellulose nanocrystals modified with profragrance molecules released the fragrance in a sustained manner in response to the gradual acidifying of pH.^[^ ^48^ ^]^ The odor, released as a volatile compound, was sensed by dynamic headspace analysis. Therefore, this strategy, which makes it possible to control the generation of volatile compounds, has been used to detect drinking water contamination. Gold NPs functionalized with a profragrance molecule (succinic acid ester of phenylethyl alcohol) were used to detect bacteria based on the mechanisms used in the human olfactory system.^[^ ^47^ ^]^ In addition to cationic gold NPs functionalized with a profragrance molecule, this system also includes the lipase enzyme, which digests the profragrance and produces fragrance. In the absence of bacteria, gold NPs bind to lipase, inactivate it, and prevent the generation of scent; whereas, in the presence of bacteria, the NPs preferentially attach to bacteria and the free lipases cleave profragrance to produce a rose fragrance. Thus, drinking water contamination could rapidly be detected by the human sense of smell. In previous studies, a similar strategy was used for the detection of different types of bacteria, where the *β*‐galactosidase (*β*‐gal) enzyme was used instead of lipase.^[^ ^49^ ^]^ In the presence of bacteria or bacteria‐produced analyte, cationic gold NPs, which captured and inactivated the *β*‐gal enzyme, preferably attached to bacteria surface or their analyte through electrostatic interaction and released the *β*‐gal enzyme; the free *β*‐gal activated and catalyzed the hydrolysis of its desired substrate.

All bacteria have distinct vibrational properties that can be subjected to identifying their structures, mechanical characteristics, and identities^[^ ^50^ ^]^ The bacterial vibration is in the range of hundreds of megahertz to gigahertz and hence, the measurement of their vibration modes is challenging. This range of vibration is too high/low for the current nanomechanical resonator/Raman spectrometry tools.^[^ ^50^, ^51^ ^]^ Ultrahigh frequency optomechanical disk resonator was developed to assess the low‐frequency vibration of a single *Staphylococcus epidermidis* bacterium. More specifically, the *S. epidermidis* bacterium was placed on an ultrahigh‐frequency optomechanical disk resonator by electrospray ionization system and the frequencies of mechanical radial breathing modes were assessed before and after bacterial placement. Using theoretical framework, low‐frequency mechanical resonances and/or vibrational spectrometry of *S. epidermidis* bacterium was calculated at a single entity level. This optomechanical sensor provided valuable information on the bacteria vibration mode and gave out the vibrational fingerprint of each bacterium.^[^ ^15^ ^]^

The development of nanotechnology‐based sensors for bacterial identification and discrimination improved our ability for sensitive, selective, and rapid detection of bacteria. However, the major challenge for these approaches is their inability to robustly detect a low concentration of bacteria in biological fluids (e.g., serum, plasma, and cerebrospinal fluids). In addition, a considerable portion of the current literature ignored the specificity of the nano‐based sensors in the detection and discrimination of specific types of bacterium from other bacteria in a mixture solution. Therefore, more studies need to be conducted on the design and development of novel sensors to identify and discriminate bacteria in biological fluids. Development of such sensors will provide a significant clinical value for the early detection of infectious diseases.

As rapid and sensitive detection of drug‐resistant/susceptible bacteria can be considered as a critical step to tailor effective antibacterial agents against the desired bacterial infections, nano‐based technologies can be used for fast and accurate detection of resistance bacteria in both in vitro and in vivo environments. Currently, the drug resistance/susceptibility of bacteria are commonly being assessed by turbidity method in which the turbidity of bacterial culture is assessed in the presence of different kinds/doses of antibacterial agents. Although this traditional method is sensitive and widely used in clinic, their synergistic roles with nano‐based technologies may improve their sensitivity and detection timing.

## 3 Mechanistic Insight into Antibacterial Effects of NPs

**Figure 3:**
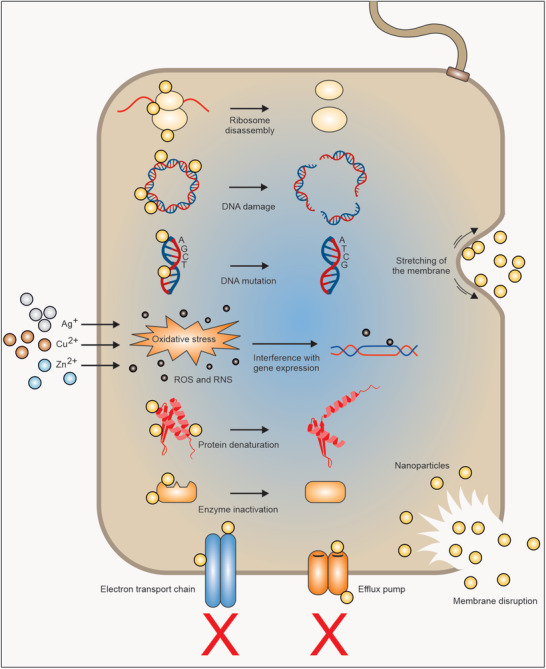
Mechanistic insight into antibacterial effects of NPs.

NPs exert their antibacterial impacts via multiple mechanisms. They can attach to the bacterial surface through several types of interactions, including electrostatic, hydrophobic, van der Waals, receptor–ligand interactions, or cross the bacterial membrane and accumulate inside bacteria.^[^ ^60^ ^]^ NPs can mechanically kill bacteria through lethal stretching of bacterial membrane.^[^ ^61^ ^]^ After adhesion to the bacterial surface, they can tear membraned and/or interfere with an electron, ion, and nutrient transport/balance, respiratory function, and efflux pump. Once inside bacteria, NPs can induce oxidative/nitrosative stress and generate reactive oxygen species (ROS)/reactive nitrogen species(RNS), damage DNA and affect gene expression, and inactivate the crucial proteins and enzymes involved in various signaling pathways. They can also damage/kill bacteria by releasing toxic ions from a distance (**Figure** **3**). Many types of NPs with different properties have been designed to kill specific bacterial species. Depending on their physicochemical properties (e.g., size, shape, surface chemistry, hydrophobicity/hydrophilicity), concentration, and incubation conditions/time, NPs show different antibacterial efficacies/impacts. In addition, the growth rate, species, wall structure, genetic background, and adaptation capability of bacteria determine their susceptibility and resistance to NPs.^[^ ^2^, ^3^, ^62^ ^]^ The effects of these parameters on NP–bacteria crosstalk and nanotechnology‐based strategies developed for bacterial killing or growth inhibition are reviewed in detail elsewhere.^[^ ^63^ ^]^ Therefore, we do not describe many examples of NPs synthesized for killing bacteria. Instead, we discuss the challenges and opportunities associated with the application of nanotechnology‐based approaches against bacteria.

## 4 Nanotechnology‐Based Biofilm Eradication

Most infectious bacteria grow as densely packed communities, sticking to each other or surfaces, and are protected by a self‐produced extracellular matrix consisting of protein, lipid, nucleic acid, and polysaccharide.^[^ ^64^ ^]^ The biofilm architecture comprises fibrillar proteins and hydrophilic polysaccharides, which keep its structure sturdy and moist, respectively.^[^ ^65^ ^]^ Extracellular polymeric substances (EPSs) act as a shield protecting the embedded bacteria from the host immune system, antibiotics, and other antibacterial agents.^[^ ^66^ ^]^ Functional and structural bacterial communities whose surfaces are protected with biofilm can endure bactericide concentrations 100–1000 times greater than suspended bacteria.^[^ ^67^ ^]^ Biofilm‐producing bacteria (e.g., *E. coli*, *Staphylococcus*, *Pseudomonas*, etc.) can migrate, colonize, and form biofilm on different surfaces of human tissues and implants, and are associated with a broad range of infections.

### 4.1 Biofilm Challenges and Resistant Mechanisms

Due to their complex structure, heterogeneity, variability, adaptive ability, and dynamic behavior, biofilms present many challenges to therapeutics; most therapeutic strategies developed to treat biofilm‐related infections have failed in clinical translation. Conventional therapeutic strategies used to date for biofilm disruption are limited by high cost, narrow host range, and low effectiveness. Therefore, the killing of biofilm bacteria is a challenging issue that requires greater attention.

**Figure 4:**
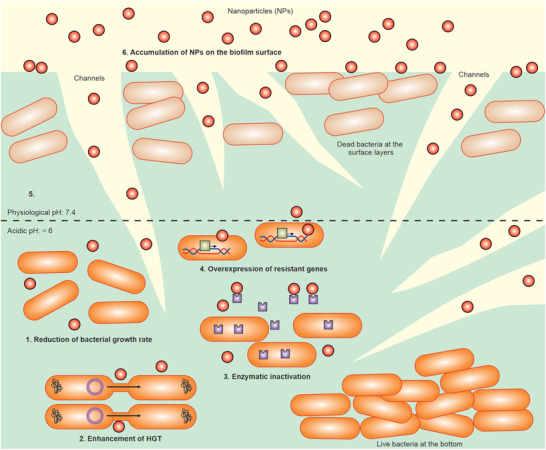
Mechanisms of biofilm‐producing bacteria resistance to NPs. Biofilm EPS acts as a barrier preventing NP access to bacteria embedded in biofilm depth. The anaerobic and acidic nature of biofilm also neutralize antibacterial agents. The biofilm‐producing bacteria use different lines of defense and strategies to protect themselves from NPs penetrating inside the biofilm. They overexpress the resistance genes and enzymes involved in NP inactivation, enhance horizontal gene transfer, and reduce growth rate.

Biofilm‐producing bacteria use different defense lines and strategies to protect themselves from NPs (**Figure** **4**). EPS is the first line of defense system preventing NP access to biofilm‐embedded bacteria. Although the biofilm structure includes channels for bacterial feeding and excretion of waste, therapeutics with large size and low infiltration have limited access to bacteria buried under layers of EPS.^[^ ^68^ ^]^ Many therapeutics are also ineffective even when they penetrate, because of the anaerobic and acidic nature of biofilm.^[^ ^2d^ ^]^ The therapeutics is currently available for the treatment of infections that killed only the bacteria residing in the outermost layers of the biofilm and had insignificant effects on deeply buried bacteria.^[^ ^69^ ^]^ In response to penetration/attack by therapeutics or stress conditions, the metabolic state of the bacterial community residing within the biofilm changes to produce enzymes that deactivate drugs through hydrolysis and/or modification. In addition, the bacterial growth rate is reduced while the expression of genes involved in bacterial resistance is enhanced.^[^ ^70^ ^]^ There is a frequent horizontal transfer of genes conferring resistance, virulence, and pathogenicity between the biofilm‐resident bacteria and those in close contact with them.^[^ ^71^ ^]^ Therefore, many bacteria embedded in biofilms are not only pathogenic and resistant to antibacterial agents, but also have undergone special adaptations to allow them to survive and spread. The main protective structure of the biofilm remains even after bacterial death, in turn triggering further attachment and colonization by bacteria.^[^ ^68^ ^]^ In addition, bacteria embedded in biofilms can move to places adjacent to the primary biofilm and form new biofilm structures.^[^ ^70a^ ^]^ The EPS regulates the osmotic gradient in the biofilm and directs the spreading of biofilm‐related bacteria.^[^ ^72^ ^]^ The biofilm‐resident bacterial community can include many bacterial species that respond differently to therapeutics.^[^ ^73^ ^]^ In addition, depending on bacterial types and communication, surface properties, and interplay with environmental factors, different types of biofilms show unique response/resistance to therapeutics.^[^ ^64^, ^65^ ^]^ Therefore, all the factors mentioned above should be considered in development of an effective therapeutic approach to eradicate biofilm‐related infections.

### 4.2 NPs against Biofilms

NPs are able not only to penetrate bacterial biofilms but also disrupt the biofilm matrix or inhibit the biofilm formation in the first place.^[^ ^70^, ^74^ ^]^ NPs can access bacteria enveloped within a biofilm and disrupt bacterial membrane integrity, induce oxidative stress, deactivate critical proteins, enzymes, and DNA, and interfere with the electron transport chain.^[^ ^66^, ^70^, ^75^ ^]^ However, NPs face many challenges in crossing the biofilm matrix and reaching embedded bacteria. Depending on the surface chemistry, attached ligands, and NP composition, NPs can interact and bind to the biofilm surface through hydrophobic, steric, or electrostatic interactions, all of which can help NPs penetrate the biofilm.^[^ ^60b^ ^]^ NP penetration of the biofilm matrix and its subsequent bacterial toxicity are also dependent upon the biofilm composition. This can include variations in bacterial channel density and pore size, thickness, and composition of EPS and the heterogeneity and density of enveloped bacteria.^[^ ^64^, ^76^ ^]^ Therefore, identical NPs may have varying effects on different biofilm‐mediated infections. As previously mentioned, on the other hand, it is also well recognized that the physicochemical properties of NPs, such as composition, size, shape, and surface chemistry/charge, are crucial for penetration and contact‐killing capability.^[^ ^2^, ^4^, ^77^ ^]^ Small‐sized NPs with hydrophobic surface chemistry easily penetrate the biofilm matrix and effectively kill deeply buried bacteria.^[^ ^78^ ^]^ The NP surface is immediately covered by biomolecules upon exposure to biological media, together called the biomolecular corona.^[^ ^79^ ^]^ The corona changes the biological identity of NPs and determines their antibacterial impact.^[^ ^80^ ^]^ Thus, the formation of biomolecular corona on the NP surface in fluid media around or within a biofilm is another issue to be considered in pursuing strategies for treating biofilm‐related infections. A few approaches to minimize the formation of protein corona at the surface of NPs^[^ ^176^ ^]^ include the use of zwitterionic coatings.^[^ ^81^ ^]^ Therefore, developing new strategies that either minimize the formation of protein corona (e.g., with the use if zwitterionic coatings) or control its composition (e.g., through a precoating approach^[^ ^82^ ^]^) may address the predetermined targeting issues of NPs.

**Figure 5:**
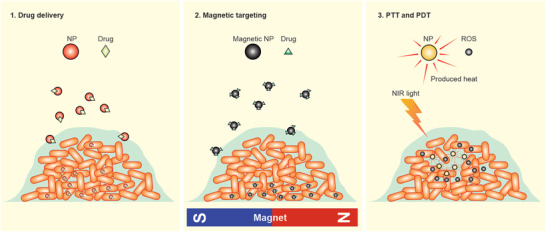
Nanotechnology‐based approaches developed for biofilm bacteria eradication. NPs are widely used for the delivery of antibacterial agents (drugs) to biofilm‐producing bacteria. Magnetic NPs are pushed inside biofilm using an external magnetic field for drug delivery to deeply bury bacteria and/or to construct additional/new channels facilitating drug delivery. Under near‐infrared (NIR) irradiation, NPs kill biofilm‐producing bacteria through photothermal therapy (PTT) and photodynamic therapy (PDT).

To prevent biofilm formation, medical devices can be covered with NPs with antibacterial and antibiofilm properties.^[^ ^83^ ^]^ However, long‐time exposure to antibacterial agents may make biofilm‐producing bacteria resistant. Therefore, it is necessary to develop new nanotechnology‐based approaches to fight against preformed drug‐resistant biofilms, not only to eradicate the biofilms themselves, but also to prevent colonization by biofilm‐producing bacteria and formation of new biofilm structures. Advanced nanotechnology‐based approaches developed for the treatment of biofilm‐related infections have included drug delivery to bacteria residing inside biofilms, photothermal and photodynamic therapy and enhancement of magnetic NP penetration within a biofilm, and creation of artificial channels in a biofilm (**Figure** **5** and **Table** **2**); these advanced strategies are discussed below.^[^ ^75^, ^84^ ^]^

**Table 2:** Nanotechnology‐based approaches for biofilm eradication

NP composition	NP type	Biofilm bacteria	Mechanism of action	NP properties	Remark	Ref.
PEG–PLGA	Polymer	*S. aureus* and *P. aeruginosa*	Delivery of both rutin and benzamide	285 nm	Against a wide range of bacteria	^[^ ^86^ ^]^
Phosphorylcholine‐based Polymer Encapsulated Chitosan NPs	Polymer	*S. aureus* ATCC12600^GFP^	Delivery of triclosan	≈70 nm	Nanocarriers with less positive charge are more effective carriers for drug delivery to bacteria residing in biofilm depth	^[^ ^77e^ ^]^
Amphiphilic diblock copolymer, poly(ethylene oxide)‐*b*‐PCouNO (PEO‐*b*‐PCouNO)	Polymer	*P. aeruginosa*	Codelivery of nitric oxide and ciprofloxacin	10–20 nm	NO‐releasing micelles release NO under visible light irradiation	^[^ ^118^ ^]^
A dual corona vesicle (PEO‐*b*‐PCL)	Polymer	*S. aureus* *E. coli*	Delivery of ciprofloxacin and intrinsic bacteria killing property. Acts as a “poisoned sword.”	230–315 nm	PEO‐*b*‐PCL eradicated biofilm in rat model of periodontitis	^[^ ^92^ ^]^
TPGS–PLGA hybrid NP	Polymer	*P. aeruginosa*	Targeted delivery of azithromycin	**–**	TPGS is cleaved by esterase enzyme produced in biofilm and payload release in a sustained manner	^[^ ^94^ ^]^
pH‐responsive polymer NP carriers	Polymer	*S. mutans*	Delivery of farnesol and thonzonium bromide	7.7–106 nm	pH‐responsive polymer NP carriers have high drug loading capacity	^[^ ^95^ ^]^
Nanocomposite consisted of mesoporous polydopamine l‐Arg‐modified and photosensitizer indocyanine green (ICG)	Polymer	*S. aureus*	PDT, low temperature PTT, and NO release under NIR irradiation	287 nm	This nanocomposite is biocompatible and caused low‐temperature PTT having no adverse effect on normal tissues/cells	^[^ ^84c^ ^]^
Lipid–polymer hybrid NP (PLGA/DOTAP)	Lipid–polymer	*S. aureus* *P. aeruginosa* *E. coli* *S. thermophilus*	Delivery of different types of antibiotics such as rifampicin, kanamycin A, ampicillin, amoxicillin, d‐cycloserine	100–130 nm	PLGA/DOTAP prevents enzymatic malfunction of loaded antibiotics	^[^ ^88^ ^]^
Hydroxyapatite NPs	Ceramic	*P. aeruginosa*	Delivery of polymyxin B antibiotic	Depending on the bacteria extract used for NP synthesis 2–5 nm 50–60 nm 75–100 nm	Hydroxyapatite NPs synthesized in the presence of extracts obtained from different bacteria and were nontoxic to human cells	^[^ ^119^ ^]^
Silver lipoate nanocluster	Metal	*S. aureus*	Silver lipoate nanocluster modified with a molecule with a *β*‐lactam backbone (6‐aminopenicillanic acid,) ruined bacteria wall and prevented bacterial wall synthesis	3–6 nm	Silver lipoate nanocluster and 6‐aminopenicillanic acid showed synergic effects on the biofilm	^[^ ^120^ ^]^
Magnetic iron oxide NPs	Metal	*S. aureus*	Pushing magnetic iron oxide NPs within biofilm by external magnetic field	11 nm	NPs destroyed biofilm through mechanical disruption and hyperthermia	^[^ ^121^ ^]^
Magnetic‐iron oxide NPs	Metal	*S. aureus*	Creation of additional artificial channel by magnetically forcing iron oxide NPs within biofilm Enhanced delivery of gentamicin	278 ± 61 nm	The artificial channels dug by magnetic iron oxide NPs facilitate antibiotic delivery to bacteria embedded in biofilm	^[^ ^104^ ^]^
Magnetic NPs	Metal	*S. aureus*	Magnetically forcing homogenous distribution of iron oxide NPs into biofilm Delivery of gentamicin antibiotic	60 nm	Homogenous distribution of NPs was occurred after 5 min treatment with external magnetic field	^[^ ^106^ ^]^
Magnetic Galinstan‐based liquid‐metal droplets (≈200 nm to ≈2 µm)	Metal	*P. aeruginosa* and *S. aureus*	Droplet force and sharp edge resulted from GLM–Fe exposure to magnet destroyed the bacterial membrane and biofilm	≈200 nm to ≈2 µm	GLM–Fe killed all bacteria embedded in biofilm after 90 min exposure to magnet	^[^ ^109^ ^]^
Cu_9_S_8_ NPs covered with PEG	Metal	*S. aureus*	PTT and PDT under NIR irradiation	190–220 nm	Synergic results of PTT and PDT effectively destroy implant‐related bacteria	^[^ ^84b^ ^]^
Gold NPs modified with DNase enzyme	Metal	*S. aureus* *P. aeruginosa*	PTT, PDT, and enzymolysis under NIR irradiation	2.3 nm	DNase enzyme destroys EPS, facilitating NP access to bacteria	^[^ ^102^ ^]^

DOTAP, Dioleoyl‐3‐trimethylammonium propane.

### 4.3 Nanotechnology‐Based Drug Delivery for Killing of Bacterial Biofilm

The critical clinical challenge for killing biofilm‐related bacteria is penetrating the EPS and delivering the desired concentration of drugs to the deeply buried bacterial community. Nanosized carriers are superior tools to load/encapsulate, transport, and deliver antibacterial agents as well as other drugs having synergic effects. The safety and therapeutic efficacy of the NPs are highly dependent to the interplay between their physicochemical, biological, and nano‐biointerface parameters which have been comprehensively reviewed recently.^[^ ^85^ ^]^ To eradicate biofilm infections, one must not only kill bacteria but also prevent biofilm formation. The simultaneous release/delivery of drugs for these two purposes is a promising strategy to eradicate biofilm‐related infections and is an excellent application for nanotechnology‐based drug delivery platforms capable of delivering drugs in a spatiotemporal manner. Polyethylene glycol (PEG)–poly(lactic‐*co*‐glycolic acid) (PLGA) NPs loaded with both rutin and benzamide successfully killed biofilm‐related bacteria and prevented new biofilm formation.^[^ ^86^ ^]^ Rutin is a natural compound that inhibits biofilm formation, while benzamide is an antibiotic effective against a wide range of bacteria.^[^ ^87^ ^]^ PEG–PLGA NPs loaded with both rutin and benzamide penetrated biofilm, released both drugs in a sustained manner, disrupting the membrane integrity of the biofilm‐forming bacteria. This drug‐loaded nanocarrier not only killed pathogens but also prevented the formation of new biofilms.

Polymeric and liposomal NPs are widely used for the delivery of antibacterial agents. Polymeric NPs can release the loaded drugs in a sustained manner, whereas liposomes can merge with the bacterial membrane and directly deliver the payload inside bacteria.^[^ ^2^, ^84^ ^]^ The hybridization of polymeric/liposomal NPs is an effective strategy to obtain synergic effects of both components to increase drug loading, enhance penetration within a biofilm, and improve bacterial targeting and drug release. Liposomal‐based drug formulations have also been extensively used in a clinical setting due to their enhanced bioavailability and greater localized drug release. High molecular weight therapeutics and hydrophobic drugs are popular therapeutic candidates for liposomal‐based NPs due to their ability to be encapsulated within a hydrophobic core. A lipid–polymer hybrid NP (LPN) consisting of PLGA/DOTAP was formulated to deliver wide‐spectrum antibiotics to deeply buriedbacteria.^[^ ^88^ ^]^ The LPN penetrated across biofilm, shielded the loaded drug from enzyme modification and consequent malfunction, attached to both gram‐negative and gram‐positive bacteria embedded in biofilm, and released loaded antibiotics in a sustained manner. The loaded antibiotic killed biofilm bacteria at a concentration 32‐fold lower than its free counterpart with the carrier.

The majority of nanocarriers developed for targeting biofilm‐related infections have a positive surface charge. Although positively charged nanocarriers apparently have a strong electrostatic interaction with bacteria inside biofilms due to their negatively charged membrane, they are usually entrapped in the EPS matrix and cannot fully penetrate the biofilm.^[^ ^84^, ^89^ ^]^ In some cases, however, the EPS can affect the NP surface charge and stability. For example, various concentrations of EPS (10–250 mg L^−1^) could decrease the surface charge of silver NPs up to −30 MV. The strong negatively charged NPs could then repel each other through electrostatic forces and, therefore, show more colloidal stability.^[^ ^90^ ^]^ Therefore, to improve penetration efficacy, we must regulate the NP surface charge. A nanocapsule consisting of chitosan (CS), a polycationic polymer, and poly(2‐ methacryloyloxyethyl phosphorylcholine) (PMPC), an amphiphilic polymer, was synthesized for delivery of antibacterial drugs into the depth of a biofilm.^[^ ^77e^ ^]^ The authors demonstrated the regulation of surface charge during nanocapsule synthesis. Therefore, identical nanocapsules with varying positive charge values were created, and those with a less positive charge achieved greater biofilm penetration. The chitosan core of this nanocapsule was loaded with antibacterial drugs and released them in a sustained manner when exposed to acidic pH and subsequent protonation of amino groups. The triclosan‐loaded PMPC–CS rapidly passed the EPS matrix and targeted the bacteria; the acidic environment of the biofilm caused the chitosan to swell, releasing the drug and killing bacteria.

Multifunctional carriers with intrinsic antibacterial activity have also been developed to deliver antibiotics to biofilms, specifically within the oral cavity. Oral biofilms are associated with infections such as periodontitis, which affect most of the world's population.^[^ ^4a^ ^]^ In periodontitis, the biofilm formed on the surface and roots of teeth destroys periodontal connective tissue.^[^ ^91^ ^]^ A dual corona vesicle consisting of poly(*ε*‐caprolactone)‐*block*‐poly(lysine‐*stat*‐phenylalanine) [PCL‐*b*‐P(Lys‐*stat*‐Phe)] and poly(ethylene oxide)‐*block*‐poly(*ε*‐caprolactone) [PEO‐*b*‐PCL] was formulated for delivery of ciprofloxacin (CIP) to biofilm bacteria.^[^ ^92^ ^]^ The PEO vesicle enhances the diffusion rate of the resulting complex, whereas P(Lys‐*stat*‐Phe)] has bactericidal effects. This multifunctional carrier rapidly passed the EPS, targeted bacteria, released antibiotics, and destroyed biofilm at only half the normal effective concentration of CIP. The authors called the CIP‐loaded dual corona vesicle a “poisoned sword” for the synergic antibacterial ability of both carrier and loaded drug.

Although a pH‐sensitive carrier with surface‐charge‐adaptive capability is a promising candidate to bypass the biofilm barrier and deliver antibiotics to bacteria, it is not effective for eradication of biofilm‐generating infections in the lungs of cystic fibrosis (CF) patients, because during aerosol administration, the NP surface charge changes before it reaches the target bacteria.^[^ ^89^, ^93^ ^]^ A hybrid of PLGA and d‐*α*‐tocopheryl polyethylene glycol 1000 succinate (TPGS) was fabricated to target delivery of azithromycin to biofilm infections in the lung.^[^ ^94^ ^]^ PEG is commonly used to prevent protein attachment (i.e., corona formation) on the surface of NPs and can stabilize the surface charge. TPGS is cleaved explicitly by the esterase enzyme produced by biofilm‐related bacteria. This hybrid NP easily diffused within the EPS and released payload upon exposure to bacteria‐produced esterase and eradicated the biofilm formed by *P. aeruginosa* in the lungs of CF patients. For another pH‐responsive polymer (poly(dimethylaminoethylmethacrylate)‐*b*‐poly(dimethylaminoethyl methacrylate‐*co*‐butyl methacrylate‐*co*‐propylacrylic acid)), NP carriers were also developed for delivery of thonzonium bromide and farnesol to biofilm‐producing *Streptococcus mutans*.^[^ ^95^ ^]^

Cinnamaldehyde is a U.S. food and drug administration (FDA)‐approved food flavoring compound that has strong antibacterial and antifungalproperties.^[^ ^96^ ^]^ However, its clinical application is restricted due to its instability and low water solubility. These challenges were overcome after immobilization of cinnamaldehyde on the surface of gold NPs. The cinnamaldehyde gold NPs could damage both gram‐negative and ‐positive biofilm‐producing bacteria and inhibited biofilm formation.^[^ ^97^ ^]^ In a similar study, silica‐coated gold NPs were used for the delivery of cinnamaldehyde to biofilm producing bacteria. This type of NPs could also inhibit biofilm formation in both gram‐negative and ‐positive bacteria.^[^ ^98^ ^]^

### 4.4 Photothermal and Photodynamic Therapy

Photothermal therapy (PTT) and photodynamic therapy (PDT) based on NPs are effective noninvasive and controllable strategies, with high selectivity and low toxicity, to kill biofilm‐related bacteria through localized hyperthermia and induction of oxidative stress (respectively) under near‐infrared (NIR) irradiation.^[^ ^84b^ ^]^ These approaches effectively destroy implant‐related biofilm infections, which are persistent problems in dental and orthopedic clinics. Using these strategies, it is possible to damage bacteria residing in biofilms without inducing drug resistance. Biofilm‐producing bacteria tend to attach to the implant surface, colonize, and then form a biofilm. Cu_9_S_8_ NPs covered with PEG have excellent PTT and PDT properties under NIR irradiation.^[^ ^84b^ ^]^ When exposed to an 808 nm NIR laser, they eradicated *S. aureus*‐produced biofilm formed on the surface of titanium (Ti) plates by synergic localized heating and induction of oxidative stress, leading to the production of ROS. Alone, neither PTT nor PDT is sufficient to kill biofilm‐related bacteria. Therefore, they are used in a synergistic manner or as supportive antibacterial agents in a multifunctional nanocomposite.

Although the synergistic combination of PTT and PDT is effective for the disruption of biofilm, the high local temperature (≈50 °C) produced by local heating can damage the normal cells/tissues surrounding the biofilm.^[^ ^99^ ^]^ Therefore, it became necessary to develop NPs capable of killing bacteria through low‐temperature PTT. Mesoporous polydopamine (MPDA) functionalized with l‐Arg‐modified, and photosensitizer indocyanine green (ICG) destroyed biofilm bacteria through low‐temperature PTT (<45 °C) and PDT under NIR irradiation.^[^ ^84c^ ^]^ The A‐ICG–MPDA nanocomposite produced local heat and ROS, inducing the generation of nitric oxide (NO) gas from l‐argentine (as a NO donor) under UV irradiation. NO prevented bacterial colonization and biofilm formation by damaging bacterial DNA and membrane. It interacts with ROS and produces reactive peroxynitrite, inducing lipid peroxidation and membrane disruption. Moreover, it is well recognized that NP‐induced PTT and PDT are much more effective when the NPs easily penetrate the EPS and access bacteria deeply buried in biofilm.^[^ ^70^, ^100^ ^]^ Scavenger enzymes such as DNase can disrupt EPS and the biofilm structure to prevent bacterial colonization and consequent biofilm formation.^[^ ^101^ ^]^ DNase‐conjugated gold NPs impaired the EPS, disrupted biofilm integrity, and killed bacteria (both gram‐negative and gram‐positive) through PTT, PDT, and enzymolysis under NIR irradiation.^[^ ^102^ ^]^ These novel NPs were used to successfully eradicate the biofilm infection formed on orthodontic devices (i.e., invisalign tooth aligners). Therefore, the combination of PDT and PTT with enzymatic digestion is a promising approach to eradicate the biofilms formed on implantable devices. By replacing the DNase enzyme with other scavengers such as proteinase and RNase, it is possible to fabricate different therapeutics for treating biofilm infections.

### 4.5 Magnetic‐Based Killing of Biofilm Bacteria

Low diffusion and accumulation of antibacterial agents in the outer layers of biofilms are the biggest challenges in treating biofilm infections. Therefore, it is necessary to develop strategies to enhance drug/NP penetration across EPS. New strategies based on magnetic targeting of antibacterial and antitumor agents are now widely used for the treatment of infections and cancers.^[^ ^103^ ^]^ Magnetic NPs (MNPs, either bactericidal themselves or carrying a bactericide) are driven directly into the biofilm's depth using an external magnetic field and provide an additional noninvasive approach to kill bacteria. In addition, they can also be used to construct new channels to facilitate the diffusion of drugs across the biofilm as magnetic NPs migrate along the external magnetic field.^[^ ^104^ ^]^ This strategy could render the biofilm more sensitive to even conventional therapeutics. However, homogenous distribution of magnetic NPs within a biofilm will generate resembling channels in biofilm, which guarantees delivery of effective concentrations of bactericide to all bacteria, regardless of the portion of the biofilm architecture they reside in, and avoid bacterial resistance.^[^ ^105^ ^]^ Iron oxide (Fe_2_O_3_) NPs, a commonly utilized magnetic NP due to their super‐paramagnetic properties, conjugated to gentamicin, showed homogenous penetration of a *S. aureus* biofilm, and killed the bacteria localized at the bottom of the biofilm when exposed to an external magnetic field. The NPs showed homogenous distribution across biofilm after only 5 min exposure to the magnetic field.^[^ ^106^ ^]^ Therefore, the exposure time should be considered/optimized through the development of magnetic targeting NPs.

Increasing the number of ducts, channels, or perforations in biofilms is an efficient means to bypass the EPS barrier and improve drug penetration and to bacteria deeply buried in biofilms. The biofilm channels (typically formed to provide nutrient, water, and waste exchange) are usually generated by bacterial movement within a biofilm.^[^ ^65^, ^107^ ^]^ For example, the additional channels formed by the motion of *Bacillus* swimmers make the biofilm structure quite sensitive to antibacterial agents.^[^ ^108^ ^]^ This suggested that the approach of increasing the number of channels by magnetically pushing NPs across the biofilm is efficacious. Under exposure to an external magnetic field, magnetic‐iron‐oxide NPs migrated along a magnetic field and across the biofilm to form new channels, which improved the diffusion of gentamicin inside the biofilm.^[^ ^104^ ^]^ The new channels increased the bactericidal effect of gentamicin fourfold to sixfold, demonstrating proof of concept that the creation of additional perforations effectively improves the penetration and bactericidal efficacy of drugs.

In another novel strategy, liquid metal droplets have been used for biofilm infection treatment.^[^ ^109^ ^]^ Liquid metals are those with a melting point low enough to be liquid in the environment.^[^ ^110^ ^]^ They are composed of a liquid core covered by an oxide layer of nanoscale thickness, formed through surface oxidation and ion movement into the outer layer (Cabrera–Mott oxidation process).^[^ ^111^ ^]^ The liquid metal forms nanodroplets upon exposure to room temperature. Magnetic Galinstan‐based liquid‐metal droplets (GLM–Fe) were also used for biofilm eradication by the combination with an external magnetic field.^[^ ^109^ ^]^ GLM–Fe were activated/spun and changed shape from spherical to the rod‐ and star‐shaped with sharp edges after exposure to dynamic/rotating magnets. The synergic results of droplet force resulting from NP movement/activation and bacterial membrane damage caused by the NPs’ sharp edges decomposed the biofilm matrix and killed both gram‐negative and gram‐positive bacteria. The liquid‐metal‐based eradication of biofilm infection is a novel strategy to destroy existing EPS, kill biofilm‐producing bacteria, and inhibit biofilm formation.

The killing of bacteria utilizing engineered magnetic NPs has not only been demonstrated through the physical deterioration of bacterial membranes, channels, or EPS. However, it can also be eradicated through magnetic hyperthermia, or localized heating through an external magnetic field. Under an external alternating magnetic field (AMF), NPs exhibit heating effects due to the motion of the magnetic dipole moment. There are several mechanisms by which this process occurs, but these are beyond the scope of this review. Recently, it was shown that magnetic NPs exposed to an AC magnetic field damage biofilm and lead to biofilm detachment through hyperthermia effects. NPs can also cause biofilm disruption and dispersal under a DC magnetic field.^[^ ^77e^ ^]^ Therefore, MNPs can affect biofilm integrity by different mechanisms under rotating AC and DC magnetic fields.

Furthermore, due to the noninvasive nature of magnetic stimuli and many biomedical applications of MNPs, mild magnetic hyperthermia can be a viable option for treating and preventing bacterial infection within biological systems. In a recent study, iron oxide NPs in the presence of an AMF‐induced mild magnetic hyperthermia (i.e., temperatures below possible tissue injury) increase the susceptibility of *Staphyloccocus aureus* to conventional antibiotics.^[^ ^112^ ^]^ Nonetheless, the concentration of treated iron oxide NPs, strength of AMF, and duration of AMF exposure are all essential parameters to consider when designing an antibacterial therapy for biological systems. Another study demonstrated that the use of iron oxide NPs coated with a block copolymer [i.e., poly((oligo(ethylene glycol) methyl ether acrylate‐*block*‐poly(monoacryloxy ethyl phosphate))] induced magnetic hyperthermia, biofilm detachment, and subsequent bacteria killing (*P. aeruginosa*) after treatment with an antibiotic, gentamicin.^[^ ^113^ ^]^ Polymer‐coated NPs are advantageous for enhancing NP colloidal stability, surface modification, and mitigating the formation of a NP–corona matrix in biological microenvironments.

Finally, MNPs can also be incorporated within different nanomaterials to form hybrid composites. Researchers have demonstrated the use of iron oxide (Fe_3_O_4_) NP and zinc oxide (ZnO) composite for the effective killing of *E. coli* and *S. aureus* in an effort to utilize a combinatorial effect of hyperthermia via magnetic nanomaterials combined with the antibacterial effects of zinc oxide.^[^ ^114^ ^]^ Overall, MNPs can affect biofilm integrity and increase bacterial susceptibility to therapeutics by increasing penetrance through the damaging of biofilm components as well as localized heating induced through an external magnetic field.

Recent findings revealed that iron‐based magnetic NPs can also activate the immune system to remove bacterial biofilms.^[^ ^115^ ^]^ This is, at least in part, due to the unique capacity of iron oxide NPs in changing the functionality of macrophages from M2 to M1.^[^ ^116^ ^]^ Future studies should focus on the synergistic roles of nanotechnologies and immune systems to remove/prevent bacterial biofilms; synergistic effects of NPs and the immune system may address major infectious clinical issues, including chronic wounds.^[^ ^117^ ^]^

## 5 NP Impacts on Bacterial Community in the Environment

### 5.1 Naturally Synthesized NPs

**Figure 6:**
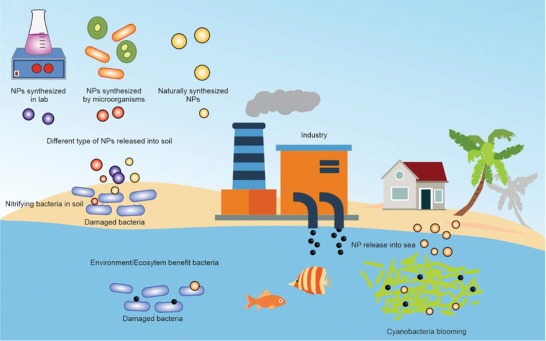
NP synthesis and release into the environment. NPs damage the environment/ecosystem (soil/aquatic) benefit bacteria and enhance *Cyanobacteria* blooming.

NPs can also be naturally generated without human intervention through biological, thermal, and photochemical processes, can be inadvertently produced in waste treatment, mines, and by an industrial process (**Figure** **6**).^[^ ^122^ ^]^ Metal NPs are naturally formed through the interaction of metal salt ions with ROS and/or natural organic matter (NOM).^[^ ^123^ ^]^ NOM is a mixed matrix consisting of biomolecules and humic substances.^[^ ^124^ ^]^ Depending on the NOM's composition and concentration, ionic strength, electrolyte content, and interaction conditions (e.g., temperature, pH), the naturally synthesized NPs (NSNPs) show different degrees of stability.^[^ ^125^ ^]^ For example, nanosized silver and gold particles can be synthesized by interaction of NOMs mainly consisting of fulvic and humic acid (HA), with ionic salts and under warm conditions. They have been found to be stable in water for an extended time and can be transported to long distances by wind and water.^[^ ^123^, ^126^ ^]^ Interestingly, the composition of the NOMs involved in NSNPs yields different antibacterial effects. For example, surface‐bound HA reduces the toxicity of hematite NPs against *Pseudomonas putida* through inhibiting NP binding to the bacterial surface and ROS generation.^[^ ^127^ ^]^

Microorganisms can act as nanofactories that capture specific ions from their environment and convert them to eco‐friendly inorganic NPs. Specifically, they can internalize ions or trap ions on their surfaces and then convert them to NPs through enzymatic processes.^[^ ^128^ ^]^ Environmental variables such as media composition, pH, temperature, ionic strength, and incubation time can affect the physicochemical properties of NPs synthesized by microorganisms.^[^ ^129^ ^]^ In some cases, the magnetic NPs generated inside bacteria play crucial roles in bacterial migration.^[^ ^130^ ^]^ As one of the most ancient biomineralizing organisms, magnetotactic bacteria have magnetosome organelle containing crystals of iron oxide NPs (magnetite), which create magnetic dipole allowing bacteria to orient in geomagnetic field.^[^ ^131^ ^]^ The empty magnetosome was observed in magnetotactic bacteria, indicating that this organelle is formed before magnetite biomineralization.^[^ ^132^ ^]^ Thanks to their ability to produce iron oxide NPs, magnetotactic bacteria can recognize both the direction and gradient of magnetic fields. Different bacterial magnetofossils produce different types of magnetites subjected for tracking the life evolution.^[^ ^133^ ^]^ Over time, as the defense systems of NP‐producing bacteria evolved, they became resistant to the biosynthesized particles and, therefore, survive even at high concentrations of metal ions and NPs.^[^ ^134^ ^]^ Although NSNPs exist naturally in soil and water, their toxic effects against eco‐friendly bacteria have not been well understood. Since NSNPs are relatively rare in the environment, scrutinizing their toxic effects on bacteria is not a priority and is overshadowed by studies of engineered NPs.^[^ ^9b^ ^]^ Although NSNPs showed less toxic effect against bacteria, their possible effects on certain microorganisms, especially those that benefit the environment and ecosystem, should be studied in future.

### 5.2 Engineered NPs

#### 5.2.1 NP Impacts on Soil Bacterial Communities

The booming application of engineered NPs in medicine, industry, and consumer products led to increased NP release into the environment during synthesis, shipping, and waste. Engineered NPs have been detected in water, sludge, waste, and soil. As a result, it is necessary to investigate their unintended effects on eco‐friendly bacteria.^[^ ^9b^ ^]^ Soil is the source of many bacteria containing antibiotic‐resistant genes.^[^ ^135^ ^]^ The release of NPs into the environment and/or coexistence of NPs with soil bacteria raise concerns about the adverse effects of NPs on the soil antibacterial resistance (Figure 6 and **Table** **3**). Rare earth oxide NPs such as La_2_O_3_, Nd_2_O_3_, and Gd_2_O_3_ increased soil resistance to tetracycline and macrolide–lincosamide–streptogramin B by enhancing the expression of antibiotic‐resistant genes and horizontal gene transfer.^[^ ^136^ ^]^

**Table 3:** NP impacts on environmental/ecosystem benefit bacteria

NPs	Bacteria	Environment	Mechanism of action	NP properties	Remark	Ref.
La_2_O_3_, Nd_2_O_3_, and Gd_2_O_3_	Soil‐borne bacteria	Soil	Enhancement of the expression of antibiotic resistance genes and horizontal gene transfer	La_2_O_3_ 25 nm Nd_2_O_3_ 35 nm Gd_2_O_3_ 27 nm	Increased bacterial resistance to tetracycline and macrolide–lincosamide–streptogramin B antibiotics	^[^ ^136^ ^]^
Ag–citr Ag–PVP Ag_2_S–PVP	*A. globiformis* *P. putida*	Soil	Disruption of membrane, respiratory chain, and biomolecules	Ag–citr 49 nm Ag–PVP 58 nm Ag_2_S–PVP 36 nm	Organic matter binding to NP surface affects NP toxicity against soil bacteria	^[^ ^138^ ^]^
Lithium intercalating compounds	*B. subtilis*	Soil	Release Ni and Co ions and damage DNA and respiration system and inducing sporulation	80–120 nm	Industrial NPs can be released into environment and produce metal ions	^[^ ^140^ ^]^
Cadmium selenide (CdSe), cadmium selenide coated with zinc sulfide (CdSe/ZnS QDs), and silicon QDs	*Shewanella oneidensis MR‐1* *B. subtilis*	Soil	Interference with respiration system and disruption of membrane integrity	4.6 nm 9 nm 4.5 nm	Silicon‐based QDs are the safest and show the least antibacterial effects against soil bacteria	^[^ ^142^ ^]^
Silver NP ZnO	Plant‐growth‐promoting bacteria	Soil	ND	11.79 ± 4.74 10 nm	Silver and ZnO NPs are bactericidal	^[^ ^144^ ^]^
TiO_2_	Plant‐growth‐promoting bacteria	Soil	TiO_2_ NPs interact with bacterial membrane and disrupt membrane integrity	46.95 ± 6.74	The formation of biomolecular corona cover on the NP surface and prevented NP–bacteria interaction and membrane damage	^[^ ^145^ ^]^
P25, anatase, and rutile TiO_2_ NPs	Sludge bacteria community	Soil/water (sludge)	Induction of oxidative stress, disruption of membrane integrity, increasing membrane permeability	P25 21 nm Anatase 10 nm Rutile 40 nm	NP‐induced bacterial death lessens sludge dewaterability	^[^ ^154^ ^]^
PVP‐coated silver NPs	Nitrifier bacteria community	Water (estuary)	Overexpression of nitric oxide reductase genes and activation of nitrogen metabolism and hydroxylamine oxidation pathway	10 nm 30 nm 100 nm	PVP‐coated silver NPs interfere with nitrification process	^[^ ^149^ ^]^
Silver NPs	Microbial plankton community *(Synechococcus)*	Coastal marine site	The released Ag^+^ ions inhibit *Cyanobacterial* growth and photosynthesis	40–80 nm	The released Ag^+^ ions may interfere with marine food chain	^[^ ^150^ ^]^
Amine‐coated polystyrene NPs (PS—NH_2_)	*M. aeruginosa* (*Cyanobacteria*)	Water	Inactivation of photosystem II, induction of oxidative stress, expression of transporter proteins, and disruption of membrane integrity	50 nm	The excessive synthesis and release of microcystin is a bacterial defense mechanism against PS—NH_2_	^[^ ^155^ ^]^
Gold–citr Gold–CTAB Gold nanospheres and nanorods	Nitrifying bacteria communities	Water	Shift in genes involved in nitrogen recycling and antibiotic/metal resistance	Nanorod 30–40 nm Nanosphere 10–20 nm	The possible antibacterial effects of NPs predicted by metagenomic analysis	^[^ ^9c^ ^]^

ND, not determined.

It is well recognized that the physicochemical properties and synthetic identity of NPs change when they are released into the environment.^[^ ^137^ ^]^ However, most studies have assessed only the antibacterial effects of the as‐synthesized NPs. Depending on the dissolved organic matter surrounding the NPs, the complexity of the exposure media, and soil pH, NPs show a wide range of stability and colloidal state in different environments. For example, silver NPs released into sewage change to Ag_2_S, which is less toxic than pristine silver NP.^[^ ^138^ ^]^ Organic matter, especially HA and fulvic acid, attach to the NP surface and reduce its toxicity against soil bacteria.

NPs’ exposure to the environment could be a threat to beneficial microorganisms. For example, lithium intercalating compounds are a new type of nanosized complex metal oxide used for energy storage in batteries.^[^ ^139^ ^]^ However, these NPs are released into the environment when the batteries are discarded. This nanocomplex releases Co and Ni ions, which inhibit the growth of soil bacteria through interference with cellular respiration, damaging DNA, and enhancing sporulation.^[^ ^140^ ^]^

Quantum dots (QDs) are another type of engineered NPs widely used for industrial products for their unique optical characteristics.^[^ ^141^ ^]^ Different kinds of QD derivatives such as cadmium selenide, cadmium selenide coated with zinc sulfide, and silicon QDs showed other toxic effects against beneficial soil bacteria.^[^ ^142^ ^]^ Cadmium selenide killed both gram‐negative and gram‐positive bacteria in a concentration‐dependent manner and via disruption of membrane integrity and respiration, while other types of QD derivatives showed negligible antibacterial effects. Therefore, silicon‐based QDs are preferred over cadmium‐based QDs for commercial products, thanks to their lower antibacterial impacts.

Plant growth‐promoting bacteria (PGPR) play a crucial role in agricultural yield by enhancing organic compound decomposition, phosphate solubilization, nitrogen fixation, and production of phytohormones, leading to plant growth.^[^ ^143^ ^]^ They also form biofilm and compete with plant pathogens to prevent their growth. Due to the risk of environmental contamination by NPs, some studies investigated the topic role of NPs on PGPR. For example, the effects of silver and zinc oxide NPs on three types of biofilm‐producing, nitrogen fixer, and phosphate solubilizer bacteria were investigated. Results revealed that the silver NPs could considerably kill the *Proteobacteria*, *Actinobacteria*, and *Firmicutes*, while zinc oxide NPs showed insignificant effects on these bacteria.^[^ ^144^ ^]^ In another study, it was shown that TiO_2_ NPs could strongly inhibit the growth of PGPR through physical interaction with bacterial membranes.^[^ ^145^ ^]^ The outcomes suggested negligible toxic effects of NPs against bacteria in soil/media rich in organic matter/biomolecules. The adsorption of organic matter/biomolecules on the NP surface can change their physicochemical properties (e.g., surface charge) and alter the NP–bacteria interaction.^[^ ^145^ ^]^

#### 5.2.2 NP Impacts on Aquatic Bacterial Communities

The release of NPs into water sources – including natural bodies such as estuaries, rivers, and oceans, as well as wastewater – and their ecotoxicological effects on aquatic microorganisms is another concern (Figure 6 and Table 3). For example, the concentration of silver NPs in the surface water is about 0.2 to 1.8 µL^−1^,^[^ ^146^ ^]^ high enough to affect bacteria beneficial to the aquatic environment. Nitrifier microorganisms, which regulate the equilibrium of oxidized and reduced nitrogen, proliferate slowly and are severely affected by NPs and other water pollutants.^[^ ^147^ ^]^ N_2_O produced during nitrification reacts with ozone and is known as one of the major causes of climate warming.^[^ ^148^ ^]^ Polyvinylpyrrolidone (PVP)‐coated silver NPs show dual effects on N_2_O production, i.e., low concentrations of NPs enhanced N_2_O production up to twofold, while high concentrations inhibited this process.^[^ ^149^ ^]^ Low concentrations of PVP‐coated silver NPs trigger N_2_O production by increasing the expression of the nitric oxide reductase gene involved in response to stress and nitrogen metabolism and activating the hydroxylamine oxidation pathway in nitrifiers. Ag+ ions mainly cause NP‐induced interference in the nitrification process; therefore, NPs released into the aquatic environment can disrupt the nitrification balance and impede the ecosystem's nitrogen cycle.

Using next‐generation sequencing, the effects of the physicochemical properties of gold NPs such as surface coatings (polyacrylic acid or cetyltrimethylammonium bromide (CTAB)) and morphology (e.g., gold nanorods and nanospheres) on nitrifying bacteria were investigated.^[^ ^9c^ ^]^ The metagenomic analysis is an advanced method of evaluating the potential effects of NPs on environmentally beneficial bacteria. This study showed that the morphology of gold NPs has a more significant effect on the functional gene profile and taxonomy/structure of nitrifying bacteria communities than does their surface coating. Gold nanorods caused the most severe shift in genes involved in nitrogen recycling and antibiotic/metal resistance, and gene‐transfer elements. The continued development of metagenomic analysis and high‐throughput sequencing of bacterial genomes is critical in designing and synthesizing environmentally friendly NPs.

The microbial plankton community and marine ecosystem are significantly influenced by environmentally relevant concentrations of silver NPs. Even a low concentration (1 ng L^−1^) of silver NPs release enough Ag^+^ ions to inhibit *Cyanobacteria* growth and photosynthesis and interfere with the marine food chain.^[^ ^150^ ^]^ The cytotoxicity of Ag^+^ ions can be attributed in part to the oxidative properties of silver, which can oxidize bacterial membranes, lipids, and intracellular proteins, preventing the widespread medical and environmental use of Ag NPs.

*Cyanobacteria* are aquatic microorganisms that play key roles in nitrogen fixing and the production of oxygen and nutrients.^[^ ^151^ ^]^ Besides their benefits, *Cyanobacteria* also produce toxic blooms that are hazardous not only to aquatic microorganisms but also to animal and human health.^[^ ^152^ ^]^ Microcystin (MC) is one of the main toxic compounds produced by *Microcystis aeruginosa* during a bloom. *M. aeruginosa* produced and secreted much higher concentrations of MC when they were treated with polystyrene NPs functionalized with amine groups (PS—NH_2_).^[^ ^153^ ^]^ These NPs suppressed the expression of proteins involved in photosystem II activation, triggered oxidative stress, damaged membrane integrity, and induced the production of transporter proteins. In response to oxidative stress caused by PS—NH_2_, *M. aeruginosa* produced MC as a defense against oxidative damage. Because of the membrane disruption and transporter protein upregulation caused by PS—NH_2_, the excess MC synthesized by *M. aeruginosa* was readily released into the aquatic environment. Therefore, NPs may cause unanticipated ecotoxicity affecting aquatic microorganisms/animals, the food chain, and human health.

## 6 NP Effects on Horizontal Gene Transfer

Horizontal gene transfer (HGT) plays a key role in bacterial evolution and adaptation to environmental stress and antibiotics. Through conjugation, the most common mechanism of HGT between bacteria,^[^ ^156^ ^]^ the conjugative plasmid replicates and transfers from donor to recipient by direct donor–recipient contact or by a fertility factor acting as a bridge connecting donor and recipient. Most resistance and virulence genes are located on plasmids, which can spread easily between bacterial populations. The widespread occurrence of conjugation is the main cause of the generation and spread of multidrug‐resistant bacteria in the environment.^[^ ^157^ ^]^ HGT occurs in all bacterial communities in biofilms, the human gut, sewage, wastewater, and soil. The plasmid harboring a resistance gene is easily transferred between bacteria even during momentary contact. The conjugation of mobile elements between bacteria provides important advantages for the evolution and survival of bacterial species; in some cases, the mobile elements become inserted into the genome and persist for generations.^[^ ^157^, ^158^ ^]^

HGT by conjugation is a paradox and costly for donor bacteria, which must consume energy to replicate and transport plasmid and synthesize the proteins involved in plasmid transport. This implies that the conjugation process has no obvious benefit for donor bacteria. In addition, rival bacteria are known as recipients, which reside in the same habitat, generate adaptation to environmental stress after receiving the plasmid. Although the newcomer plasmid facilitates the adaptation of recipient bacteria to environmental stress, they also cause an additional metabolic burden, decreasing the competitiveness of recipient bacteria after environmental stress has passed.^[^ ^157^ ^]^ Therefore, bacteria tend to minimize plasmid cost, e.g., bacteria‐harboring related plasmids use restriction–modification and Crispr–Cas mechanisms to stop HGT.^[^ ^156^, ^159^ ^]^ Therefore, depending on bacterial species, their plasmid content, and type, and environmental stress, some bacteria use a defense system to prevent conjugation.

It is well understood that decreasing the use of antibiotics alone will not be an effective strategy to prevent the evolution of antibiotic‐resistant bacteria. Such initiatives should be combined with conjugation inhibition to prevent the generation of drug‐resistant bacteria.^[^ ^160^ ^]^ To prevent the widespread dissemination of mobile elements harboring resistant genes, several compounds have been used to target recipient, donor, proteins involved in plasmid replication and transfer, and even the pilus, the bacterial appendage necessary for conjugation. Although unsaturated fatty acids effectively inhibit conjugation in the laboratory, so far, this inhibitory approach has not been used to inhibit conjugation in the natural environment.^[^ ^161^ ^]^

**Figure 7:**
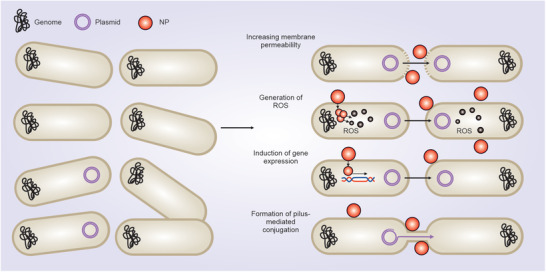
NPs enhance the horizontal transfer of conjugative plasmids between the same or different genera.

Thanks to their widespread presence in the environment, NPs may affect HGT and bacterial resistance development. Even though NPs are widely used under controlled conditions to kill drug‐resistant bacteria, NPs released into the environment enhance the HGT of conjugative plasmid and generation of resistant bacteria (**Figure** **7**).^[^ ^162^ ^]^ For example, nanoalumina enhanced the HGT of conjugative plasmids across genera (from *E. coli* to *Salmonella*) in an aquatic environment.^[^ ^162c^ ^]^ It accelerated this process up to 200‐fold by increasing membrane permeability, ROS generation, as well as up‐ and downregulating genes involved in mating pair development and regulatory conjugation, respectively. In a similar way, ZnO NPs promoted conjugation of RP4 plasmid among different genera of aquatic bacteria.^[^ ^162b^ ^]^ It was also shown that subinhibitory concentrations of CuO NPs or Cu^2+^ enhance the horizontal transfer of drug resistance genes from *E. coli* to *P. putida* via overexpressing the proteins involved in membrane permanence, pilus/mating pair formation, and induction of oxidative stress.^[^ ^162a^ ^]^ In contrast to current assumptions that NPs prevent the evolution of drug‐resistant bacteria, NPs released into the natural environment enhance the conjugative transfer of drug resistance genes across genera. The HGT of conjugative plasmids harboring drug‐resistance genes usually takes place between the same bacterial species and rarely occurs across genera.^[^ ^163^ ^]^ However, NPs do facilitate HGT of conjugative plasmids between different bacterial species.

## 7 NPs against Microbial Communication and Quorum Sensing

A “bacterial community” may consist of different bacterial species inhabiting the same location.^[^ ^164^ ^]^ They have close contact/interactions and are affected by neighboring bacteria and ecological forces based on bacterial critical needs.^[^ ^165^ ^]^ For example, their metabolic requirements are adjusted based on the metabolic function of their neighbors in the community. Bacteria living in multispecies communities have access to a wide range of metabolites and nutrients produced by their competitors and adapt to more severe ecological stress.^[^ ^164^ ^]^ Cell–cell communication through chemical‐, electric‐, and contact‐based signaling is an essential evolutionary and ecological process for the growth and development of bacterial communities.^[^ ^166^ ^]^ Depending on their species and density, as well as ecological and environmental context, bacteria in a community have different classifications and frequencies of interaction. For example, bacteria that grow in unsaturated soil have higher contact frequency compared to those growing in water‐saturated soil.^[^ ^167^ ^]^ Bacteria are social microorganisms in close contact and have inter‐ and intraspecies communications coordinating their proper response to environmental stress.^[^ ^168^ ^]^ Bacteria that grow in social communities can sense the neighboring cells and demonstrate different behaviors and capabilities compared to suspended bacteria. The molecular mechanism mediating the coordinated gene expression in bacterial communities in response to cell density and environmental stress is called quorum sensing (QS). QS comprises the synthesis, release, and sensing of threshold concentrations of autoinducers and activating the signaling pathway through changing gene regulation. Gram‐negative and gram‐positive bacteria use acyl‐homoserine lactones (AHLs) and autoinducing peptides as autoinducers, respectively.^[^ ^169^ ^]^

**Figure 8:**
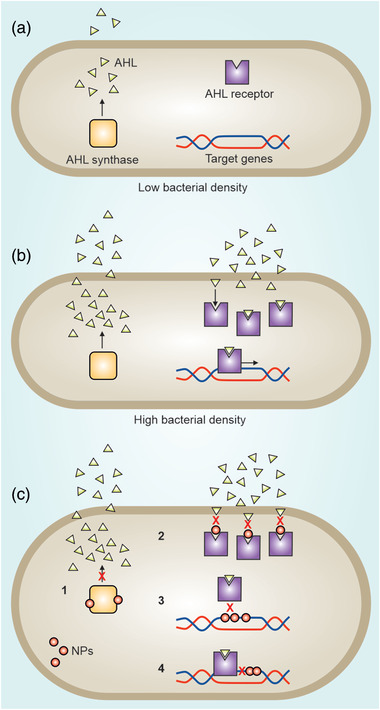
a) At low bacterial population densities, a small amount of the extracellular autoinducer produced by bacteria is dispersed away in the surrounding environment and hence, it is unable to activate the QS signal. b) Once a threshold density of bacteria has been reached (at high bacterial densities), they produce a high amount of autoinducers, which subsequently bind to receptors and activate QS signal. c) NPs inhibit QS by preventing the synthesis of autoinducer, receptor–autoinducer binding, and expression of genes involved in QS signal activation.

This density‐dependent cell–cell signaling enables bacteria to conduct activities that are energetically unfavorable in areas of low bacterial concentration, but ultimately become beneficial for the bacterial population when it reaches a critical density. As a result, bacteria constantly produce autoinducers, even at low density. The chemical signals become concentrated as the bacterial population grows. When limen density of autoinducers produced reaches a threshold, they bind to bacteria receptors and trigger the expression of genes involved in vital processes such as synthesis of biofilms, siderophores, virulence factors, and proteases, plasmid transfer, sporulation, metabolite synthesis, and adaptation to environmental stress (**Figure** **8**a,b).^[^ ^119^, ^169^, ^170^ ^]^

## 8 Mechanisms of Nanotechnology‐Based Quorum Quenching

Preventing QS is a promising approach to disarm bacteria and reduce their pathogenicity and cooperative action. Different strategies such as preventing autoinducer synthesis, enzymatic degradation of produced autoinducers, inhibition of autoinducer–receptor binding, and suppressing genes involved in signal activation have been developed to interfere with QS.^[^ ^169^, ^171^ ^]^ Although several compounds have been developed for quorum quenching, the disabling of QS has not been clinically used to treat drug‐resistant bacteria.^[^ ^171a^ ^]^

Depending on their physicochemical properties and the bacterial species, NPs are promising candidates to simultaneously prevent and/or disrupt different steps in QS signaling (Figure 8c).^[^ ^5^, ^72^ ^]^ Metal and metal oxide NPs effectively disrupted the QS signal by different mechanisms. ZnO NPs interfered with the autoinducer sensing step, whereas Ag NPs and TiO_2_ NPs prevented the generation of autoinducer in *Chromobacterium violaceum* bacteria.^[^ ^5b^ ^]^ A similar study showed that selenium NPs impede QS by preventing autoinducer synthesis, whereas tellurium NPs interfere with signal sensing and subsequent reactions.^[^ ^173^ ^]^

Although many gram‐negative bacteria have conserved QS‐based signaling mechanisms for synchronizing their social activity, they show various responses to environmental stress.^[^ ^5a^ ^]^ As each species creates different QS signals, they respond differently to identical NPs. *Pseudomonas syringae* and *Pantoea stewartii* synthesized different types of AHLs when exposed to Ag NPs and single‐wall carbon nanotube and hence, they produced other QS signals.^[^ ^5a^ ^]^ Different QS signals may cause different bacterial activities and behaviors.

QS inhibition by quorum quenchers is an effective strategy to reduce *P. aeruginosa* virulence. They suppress the production of virulence factors such as toxins, biofilms, and proteases.^[^ ^174^ ^]^ As they block only virulence factor generation and show no significant effect on bacterial growth, in most cases, quorum quenchers do not induce bacterial resistance.^[^ ^175^ ^]^ However, certain pathogenic and environmental bacteria have shown resistance to some quorum quenchers.^[^ ^176^ ^]^ ZnO NPs reduced the virulence of clinical and laboratory strains resistant to quorum quencher compounds by suppressing the expression of pyocyanin and elastase and the formation of biofilm.^[^ ^177^ ^]^ Similarly, microfabricated Ag NPs quenched QS signal in *P. aeruginosa* by reducing the expression of virulence factors and biosynthesis of AHLs and preventing the formation of biofilm.^[^ ^178^ ^]^ These reports did not investigate/report the mechanism behind inhibitory effects of the NPs in detail; therefore, further studies need to be conducted to better understand how NPs affect autoinducer synthesis/degradation, autoinducer–receptor binding, or expression of genes.

## 9 Mechanisms of Bacterial Resistance to NPs

**Figure 9:**
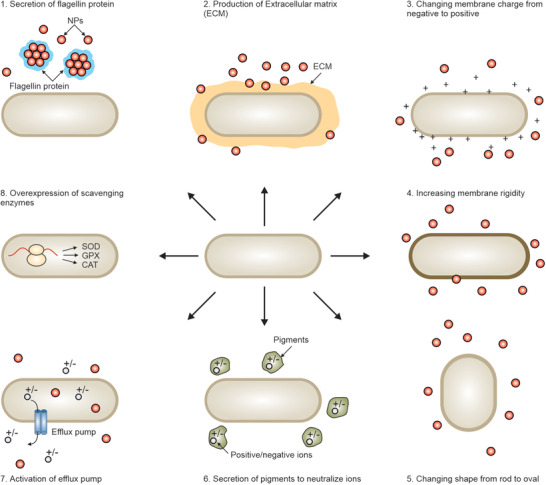
Mechanisms of bacterial resistance to NPs.

Although NPs have been introduced as an alternative to antibiotics to fight multidrug‐resistant bacteria, it is too early to expect their replacement with the current antibacterial therapeutics, as NPs may have some unexpected effects on bacteria. For example, it was shown that silver NPs cause a shift in the antibiotic resistance gene profile that may make bacteria resistant to the broad spectrum of antibiotics.^[^ ^179^ ^]^ Thanks to their genetic background flexibility and variation, bacteria are able to adapt to and endure NP‐induced stress.^[^ ^62^, ^180^ ^]^ Bacterial evolution over time has made them resistant even to new generations of antibacterial agents such as NPs. Even though NPs damage bacteria by different mechanisms, the bacteria use different lines of defense and strategies to protect themselves (**Figure** **9**). For example, efflux pumps associated with bacterial antibiotic resistance also play a role in removing toxic heavy metal ions generated from NPs. Bacteria can alter or inactivate NPs and their released ions before they reach the bacterial surfaces, prevent NP attachment to membrane, prevent NP internalization, eject internalized NPs or ions, and prevent or suppress NP‐induced oxidative stress. Bacterial defense against NPs makes use of the following mechanisms.^[^ ^6^, ^62^, ^181^ ^]^

### 9.1 Production of Extracellular Matrix/Components

As most NPs kill bacteria through disruption of membrane integrity, bacteria protect their membranes by producing the extracellular matrix (ECM), which prevents NP attachment/access to bacterial surfaces either by creating a protective shield or inducing NP aggregation. Bacteria are known to develop resistance after repeated long‐time exposure to silver NPs. *E. coli* and *P. aeruginosa* became resistant to high concentrations of silver NPs after 8 and 13 successive cultivation steps, respectively. In response to NPs, they secreted flagellin protein, which facilitated/induced NP aggregation. The agglomerated NPs (shown by transmission electron microscopy to undergo a color change from yellow to gray‐black) exerted a less toxic effect on bacteria. The most striking observation was that these bacteria became resistant to silver NPs without any statistically relevant mutation or change in their genome. Subsequent treatment of a flagellin inhibitor restored bacterial sensitivity to Ag NPs;^[^ ^6a^ ^]^ therefore, this bacterial‐resistance mechanism is temporal and activated only when they face silver NPs.

The ECM produced by bacteria consists of different polymers and biomolecules such as proteins, lipids, and polysaccharides.^[^ ^182^ ^]^ Depending on the bacterial species and stress caused by NPs, bacteria produce ECMs with different compositions and contents. In addition, the composition and concentration of ECM produced by bacteria in defense against NPs strongly depend on the bacterial growth rate, metabolic state, and environmental conditions. Because the NPs trapped in ECM are immediately covered by ECM biomolecules, the resulting corona‐coated NPs are less toxic.^[^ ^182^ ^]^ For example, the biomolecular corona can change the NP surface charge from positive to neutral or even negative, reducing NP–bacterial membrane interaction.

### 9.2 Bacterial Membrane Changes

By changing their membrane composition, bacteria can also regulate their surface charge to prevent their electrostatic interaction with positively charged therapeutics. In response to NPs, bacteria change their surface charge from negative to positive by overexpressing the enzymes responsible for synthesizing amino‐containing phospholipids. In response to therapeutics, they, respectively, up‐ and downregulate saturated and unsaturated fatty acids, leading to increased membrane rigidity and consequent reduction in membrane permeability to therapeutics.^[^ ^183^ ^]^

Bacteria can adapt themselves to NPs through acquired, intrinsic, and adaptive processes. Unlike intrinsic and acquired defenses, which are permanent and inherited by daughter cells, the adaptive defense is provisionally generated in response to NP‐induced stress.^[^ ^184^ ^]^ During the development of permanent defense against NPs, bacteria undergo genomic alteration, while for adaptive defense, the production of specific proteins changes conditionally.^[^ ^181b^ ^]^ Long‐time exposure of *E. coli* to silver NPs led to mutations in the genome inherited by subsequent generations.^[^ ^180a^ ^]^ It is well recognized that environmental stress can also temporally change bacterial shape. In the presence of ZnO NPs, scanning electron microscopy clearly showed that *E. coli* change their shape from rod to oval. The shape changes occurred by suppressing RodZ, which mediates the attachment of cytoskeleton proteins to the membrane. Moreover, there was overexpression of OmpF and OmpC, porin proteins that regulate nutrient and waste influx/efflux. All these changes were reversed after bacteria were regrown in NP‐free medium.^[^ ^185^ ^]^

### 9.3 Deactivation of Oxidative Stress and Ions Generated by NPs

NPs have been shown to induce oxidative stress, leading to excessive ROS generation, impairing enzymes, DNA, RNA, lipids, and other essential biomolecules.^[^ ^2a^ ^]^ In fact, excessive ROS kills bacteria even after removing the stressor; bacterial killing becomes self‐driven, and the self‐amplifying ROS becomes the cause rather than the consequence.^[^ ^186^ ^]^ Depending on their physicochemical properties and capability of producing ions, NPs generate different types and amounts of ROS. The most common bacterial defense mechanism against excess ROS is the upregulation of scavenging enzymes such as superoxide dismutase, glutathione peroxidase, and catalase.^[^ ^187^ ^]^ Superoxide dismutase functions to convert superoxide radicals to hydrogen peroxide and oxygen. The regulation of small molecule antioxidants in bacteria, such as glutathione or nicotinamide adenine nucleotide phosphate (NADPH), is also vital to remove ROS species.For example, *P. aeruginosa* overexpressed a superoxide dismutase gene in response to quantum‐dot‐induced oxidative stress.^[^ ^188^ ^]^ Finally, as ROS species target nucleic acid, DNA repair enzymes may also be upregulated in the presence of oxidative stress generated by NPs.

Since metal‐based NPs can damage bacteria via the release of toxic ions, several defense mechanisms have been evolved for neutralizing those ions. The first line of defense is the secretion of compounds or enzymes that inactivate ions outside the bacteria before approaching the bacterial membrane. Bacteria can secrete various components that detoxify ions via ion transformation, enzymatic detoxification, and NP aggregation, which reduce ion release by metals.^[^ ^62^, ^189^ ^]^ Bacteria can also secrete pigments that capture toxic silver ions and change them to Ag^0^, which has no antibacterial effects.^[^ ^190^ ^]^

When ions pass the bacterial membrane and accumulate inside, bacteria eject excessive ions using efflux pumps, which are often used to excrete drug compounds and ions.^[^ ^191^ ^]^ The multidrug efflux pump is a common adaptation to neutralize antibacterial agents and is found in many forms. The most clinically significant pumps are the resistance–nodulation–cell division (RND) family, which form mainly in gram‐negative bacteria and consist of an outer membrane protein (porin) and periplasmic membrane fusion protein; positively charged ions are effluxed through a substrate/H^+^ antiport mechanism.^[^ ^192^ ^]^ In response to excessive ion accumulation inside bacteria, the efflux complex protein superfamily is upregulated. For example, RND proteins are overexpressed in bacteria exposed to quantum dot ions.^[^ ^192^ ^]^

## 10 In Vivo Antibacterial Impacts of NPs

Unlike the in vitro environment, the in vivo antibacterial efficacy and side effects of NPs depend on many additional factors, including administration route, circulation time, and composition of biomolecular corona.^[^ ^80^, ^190^ ^]^ A considerable portion of the in vivo applications of antibacterial NPs has been focused on implantable biomaterials and wound healing dressings. We have recently reviewed the in vivo antibacterial effects of NPs on wound healing^[^ ^117b^ ^]^ and implantable biomaterials for bone regeneration.^[^ ^194^ ^]^ This is a section, therefore, which is focused on additional in vivo applications of antibacterial NPs.

Antibacterial NPs are being studied to remove oral bacterial biofilms (e.g., *S. mutans*), which form dental caries.^[^ ^195^ ^]^ Calcium fluoride NPs have been used to prevent *S. mutans* growth or biofilm formation on the tooth surface and therefore improve oral health.^[^ ^196^ ^]^ Calcium fluoride NPs could kill *S. mutans* and reduced its capability in acidogenesis, aciduracity, and biofilm formation in infected rat's teeth.^[^ ^196^ ^]^ These NPs may damage bacteria through direct interaction with bacterial membrane and disruption of membrane integrity or sustained release of fluoride ions which not only inhibit the virulence behaviors of *S. mutans* but also enhance the remineralization process in teeth.^[^ ^196^ ^]^ The possible roles of these NPs and others designed for oral delivery applications on gut microbiota composition must be considered in detail. It is increasingly being accepted that even small changes in gut microbiome composition and diversity can significantly affect metabolism, immune response, and nutritional digestion and trigger the development of many disorders.^[^ ^197^ ^]^ Studies revealed that NPs can change the composition of gut microbiota;^[^ ^198^ ^]^ for example, it was shown that the oral administration of TiO_2_ NPs could change the composition of gut microbiota and gut‐related metabolism in rats, which led to induction of oxidative stress and inflammation.^[^ ^198a^ ^]^ In addition, the gut microbiota imbalance caused by TiO_2_ could induce the overproduction of lipopolysaccharide.^[^ ^198a^ ^]^ A similar study showed that dietary TiO_2_ NPs interfere with the immune response by killing gut flora such as *Bifidobacterium* and *Lactobacillus*.^[^ ^198b^ ^]^

Antibacterial NPs for in vivo applications need to be safe to host cells. In this case, new compositions of NPs have been developed; for example, bimetallic Au–Ag core–shell NPs coated by carbohydrate were developed and their toxicity readouts revealed no side effects toward mammalian host cells.^[^ ^199^ ^]^ The NPs demonstrated more severe toxic effects against multidrug‐resistant (MDR) MRSA compared to antibiotics in MRSA‐infectedmice.^[^ ^199^ ^]^ Another strategy to improve the safety of NPs toward host cells is coating them with biocompatible materials. Biocompatible coatings, for example, could reduce the toxicity of silver NPs, stabilize them, and avoid NP aggregation in biological fluids such as blood. For example, pristine silver NPs showed less antibacterial performance in murine salmonellosis model compared to the silver NPs coated with poly(vinylpyrrolidone) or citrate.^[^ ^200^ ^]^ Although both coated and pristine NPs showed similar biodistribution pattern into different organs, only the coated NPs killed *Salmonella* in different organs of mouse model of infection and were safe to the healthy cells.

The use of NPs in conventional antibacterial therapy approaches could improve their antibacterial efficacy.^[^ ^2a^ ^]^ For instance, it was shown that the combination of silver NP and antibiotics (polymyxin B) could destroy the drug‐resistant *A. baumannii* in *A. baumannii*‐infected mouse peritonitis models.^[^ ^201^ ^]^ The infected mice treated with either silver NP or antibiotics showed lower survival than those treated with the NP–antibiotic combination. As another example, the addition of ZnO NPs to collagen/chitosan wound dressing nanofibers could significantly improve their antibacterial properties against *S. aureus* and *E. coli*.^[^ ^202^ ^]^ The addition of ZnO NPs to poly(vinyl alcohol) gel demonstrated effective antibacterial impacts in mice in mice elytritis (vaginitis) model infected with *E. coli*.^[^ ^203^ ^]^

NPs can also be used to deliver antibacterial drugs to the infection site of the body. For example, PLGA NPs were used as a career for delivery of antimicrobial peptides (AMPs that are capable of destroying pathogens by disrupting their membrane integrity and/or inactivation of their defense system^[^ ^204^ ^]^) to lung tissue and could effectively kill pulmonary bacteria in an animal model of *P. aeruginosa* lung infection compared to the free AMPs.^[^ ^205^ ^]^

Last but not the least, the role of sex on the safety and antibacterial properties of nanomedicine technologies needs to be considered in the in vivo impacts of NPs. This is important because recent reports revealed the critical role of sex in safety and therapeutic efficacy of nanoparticles.^[^ ^206^ ^]^

In summary, the in vivo efficacy of antibacterial NPs has been investigated mainly in wound healing dressings and implantable biomaterials. However, the critical role of biomolecular/protein corona on the safety and antibacterial efficacy of these NPs has been poorly investigated. Poor understanding of protein coronas has resulted in inaccurate safety and efficacy predictions when testing nanomedicines in animals and humans.^[^ ^207^ ^]^ We have extensively studied how protein coronas interact with NPs and regenerative medicine patches,^[^ ^80^, ^208^ ^]^ but little research has focused on protein corona formation on NPs and their antibacterial properties in vivo (e.g., for chronic wound applications). For example, although exudates in chronic wounds^[^ ^117b^ ^]^ (i.e., fluids with various types of proteins, cytokines, and other types of biomolecules) can interact with the surface of NPs and form protein corona,^[^ ^79^, ^117^ ^]^ their roles in altering the antibacterial efficacy of NPs have been poorly investigated. In this regard, we have recently probed the role of protein corona on the antibacterial properties of super‐paramagnetic iron oxide NPs, and their in vivo efficacy is a rat model of a diabetic wound.^[^ ^209^ ^]^ Our findings revealed the critical role of protein corona in altering macrophages’ proinflammatory phenotype and, therefore, their antibacterial properties against *S. aureus*. More specifically, the formation of protein corona could significantly improve the polarization of macrophages toward proinflammatory phenotype and improved their antibacterial properties.

## 11 Conclusions

Bacteria are widespread in biological systems as well as many different types of microenvironments. While some bacteria are essential for mammals to maintain homeostasis (i.e., gut microbiome) and beneficial for environmental sustainability, bacterial growth poses a serious risk during infection and presents significant challenges to address. The rapid detection of unwanted bacteria is crucial in preventing further growth. Here, this review has in part covered nanotechnology‐based approaches toward the detection of bacteria and their unique advantages. These include the versatile surface chemistry of NPs, which permit strong binding affinity between conjugated recognition elements (i.e., antibodies, aptamers, etc.) and even enable platforms that are label‐free (i.e., array‐based sensors). Furthermore, as NP‐LSPR‐based colorimetric detection methods become more well established, the future NP composition and physiochemical properties may enhance existing colorimetric methods or give researchers the tools to develop entirely new techniques.

Moreover, the treatment of bacterial infections and their resistance mechanisms to NPs are of equal importance to clinicians. Bacteria have adapted many intrinsic abilities to combat antibiotic treatments and exposure to NPs, including the secretion of NP aggregation‐inducing proteins, upregulation of multidrug efflux pumps, and expression of ROS‐scavenging enzymes. These mechanisms, as well as the components of the biofilm, have been identified as therapeutic targets in the efficient killing of bacteria, and nanotechnology has helped to both elucidate and overcome these defense mechanisms. NPs containing unique magnetic, PTT, and PDT properties can be used in conjunction with traditional antibiotics and can prove a noninvasive approach to treating bacterial infections. Looking forward, NPs and nanomaterials in general, may harbor the versatility to provide multiple methods of killing bacteria in a single platform for synergistic antibacterial therapeutics and diagnostics. Recent progress in the field of bacterial nanotechnology created unprecedented hopes among all stakeholders (especially patients) for early diagnosis and treatment of resistant infectious diseases, specifically when combined with comorbidity (e.g., chronic wounds in patients with diabetes and/or cardiac disorders). Nanotechnologies also paved the way for robust and accurate identification and discrimination of bacteria, which enable i) healthcare providers in determining the right antibiotic medications to prevent consequences of bacterial infection and ii) device developers to monitor/control pathogen spread that may be epidemic or pandemic.

NPs attack bacteria, through various mechanisms, to affect their structural integrities and functions. However, as a counter‐reaction, bacteria exert multiple mechanisms to protect themselves and resist against therapeutic NPs; such mechanisms have been poorly understood and require special consideration in future works.

Most infectious bacteria show community‐derived resistance by forming a biofilm. Due to their capability to diffuse within biofilm and access or kill the bacteria deeply buried in biofilm, it is well‐documented that NPs can be a promising candidate for destroying biofilm‐related infections. The efficiency of NPs in fighting against bacterial biofilm is strongly dependent on NPs’ characteristics (e.g., composition, size, charge, hydrophobicity/hydrophilicity) and type of bacteria.

Although NPs could be a promising alternative for antibiotics, concerns have also been raised about their unwanted adverse effects on the environment beneficial bacteria involved in public health and ecological balance. NPs’ release into the environment, which seems inevitable, may kill different bacterial communities or make them resistant. In some cases, they may enhance the horizontal transfer of conjugative plasmids harboring resistant and virulence genes. Therefore, exposure of NPs into the environment may not only kill eco‐friendly bacteria but also cause some infectious bacterial resistance that may cause unforeseen issues. Due to their rapid evolution and flexibility, bacteria have a unique capacity to adapt to their environmental stressors, which, in turn, develop resistance against NP therapeutics.

## Conflict of Interest

M.M. discloses that i) he is a Co‐founder and Director of the Academic Parity Movement (www.paritymovement.org), a nonprofit organization dedicated to addressing academic discrimination, violence, and incivility; ii) he is a Founding Partner at Partners in Global Wound Care (PGWC); and iii) he receives royalties/honoraria for his published books, plenary lectures, and licensed patents.
